# Deciphering the Hybridisation History Leading to the Lager Lineage Based on the Mosaic Genomes of *Saccharomyces bayanus* Strains NBRC1948 and CBS380^T^

**DOI:** 10.1371/journal.pone.0025821

**Published:** 2011-10-05

**Authors:** Huu-Vang Nguyen, Jean-Luc Legras, Cécile Neuvéglise, Claude Gaillardin

**Affiliations:** 1 INRA, UMR1319 Micalis, Jouy-en-Josas, France; 2 AgroParisTech, Micalis, Jouy-en-Josas, France; 3 INRA, UMR1083 Sciences Pour l'Œnologie, Montpellier, France; Duke University, United States of America

## Abstract

*Saccharomyces bayanus* is a yeast species described as one of the two parents of the hybrid brewing yeast *S. pastorianus*. Strains CBS380^T^ and NBRC1948 have been retained successively as pure-line representatives of *S. bayanus.* In the present study, sequence analyses confirmed and upgraded our previous finding: *S. bayanus* type strain CBS380^T^ harbours a mosaic genome. The genome of strain NBRC1948 was also revealed to be mosaic. Both genomes were characterized by amplification and sequencing of different markers, including genes involved in maltotriose utilization or genes detected by array-CGH mapping. Sequence comparisons with public *Saccharomyces* spp. nucleotide sequences revealed that the CBS380^T^ and NBRC1948 genomes are composed of: a predominant non-*cerevisiae* genetic background belonging to *S. uvarum,* a second unidentified species provisionally named *S. lagerae,* and several introgressed *S. cerevisiae* fragments. The largest *cerevisiae*-introgressed DNA common to both genomes totals 70kb in length and is distributed in three contigs, cA, cB and cC. These vary in terms of length and presence of *MAL31* or *MTY1* (maltotriose-transporter gene). In NBRC1948, two additional *cerevisiae*-contigs, cD and cE, totaling 12kb in length, as well as several smaller *cerevisiae* fragments were identified. All of these contigs were partially detected in the genomes of *S. pastorianus* lager strains CBS1503 (*S. monacensis*) and CBS1513 (*S. carlsbergensis*) explaining the noticeable common ability of *S. bayanus* and *S. pastorianus* to metabolize maltotriose. NBRC1948 was shown to be inter-fertile with *S. uvarum* CBS7001. The cross involving these two strains produced F1 segregants resembling the strains CBS380^T^ or NRRLY-1551. This demonstrates that these *S. bayanus* strains were the offspring of a cross between *S. uvarum* and a strain similar to NBRC1948**.** Phylogenies established with selected *cerevisiae* and non-*cerevisiae* genes allowed us to decipher the complex hybridisation events linking *S. lagerae*/*S. uvarum*/*S. cerevisiae* with their hybrid species, *S. bayanus/pastorianus.*

## Introduction

Beer and wine fermentations were acquired from the Middle East by Germanic and Celtic tribes and represent some of the most ancient fermentation technologies [1]. During the development of this technology, strains have been progressively selected for enhanced abilities according to standards for quality and production. Strains specific to beer fermentation have been isolated and studied for over one century and their diversity has led to the delineation of several species. During the 19th century ancient beers made with a mixture of yeasts were a source of yeast strains and species. In 1883, Emile Christian Hansen isolated the strain CBS1171 from beer. This strain was designated as the neo type of *Saccharomyces cerevisiae* previously described by Reess in 1870. The genus name *Saccharomyces* was first proposed by Meyen in 1838 [2]. Thereafter, many other species belonging to the genus *Saccharomyces* were isolated from beer including: *S. bayanus* (Saccardo, 1895) by Will in 1891, and *S. carlsbergensis* and *S. monacensis* by EC Hansen in 1908. Since 1888, progressively modern beers were made with pure cultures following the recommendation of EC. Hansen. Later, other sources have replaced beer as a reservoir of yeasts. For example *S. uvarum* (Beijerinck, 1898) was isolated from blackberry juice and *S. paradoxus* (Batschinskaia, 1914) was isolated from oak exudates. Forty one *Saccharomyces* species isolated between 1883 and 1965 were successively reclassified by many authors and were admitted to the genus *Saccharomyces* by van der Walt in 1970 [3]. In this new classification, *S. carlsbergensis* and *S. monacensis* described by EC Hansen, were reduced to synonyms of *S. uvarum* (Beijerinck).

However in 1985 [4], the *Saccharomyces* group known as *Saccharomyces sensu stricto* was restricted by DNA-DNA reassociation to four species: *S. cerevisiae* (neo type strain CBS1171^NT^), *S. bayanus* (CBS380^T^), *S. paradoxus* (CBS432^NT^), and *S. pastorianus* (CBS1538^NT^). These experiments also revealed that *S. pastorianus*, which includes two synonyms, *S. carlsbergensis* and *S. monacensis*, was a hybrid species with *S. cerevisiae* and *S. bayanus* as parents [4]. At the same time, *S. uvarum* (CBS395^T^) was reduced to a synonym of *S. bayanus*. Successive studies on other lager strains revealed the complexity of strains classified under the *S. pastorianus* and *S. bayanus* species. Some controversial issues appeared such as the hybrid nature of CBS380^T^ and its status of type strain. On the one hand, our previous studies revealed that *S. bayanus* CBS380^T^ was itself a chimer bearing Y' and *SUC4* sequences identical to *S. cerevisiae* sequences localized on three chromosomes [5]. This hybrid nature has been identified in other strains such as: CBS378, CLIB271, and NRRLY-1551. The latter strain is the most similar to CBS380^T^ and has been confused with CBS 1538^NT^, the current neo type strain of *S. pastorianus*. On the other hand, *S. uvarum* strains (classified as *S. bayanus*) examined in the same study lack *S. cerevisiae SUC4* and Y' sequences [6].

Three sequencing projects of the genomes from *S. uvarum* representative strains have been developed, by Broad Institute (http://www.broadinstitute.org/, [7]) using CBS7001 ( = MCYC623), by Génolevures (http://www.genolevures.org/, [8]) and the Washington University School of Medicine (http://genomeold.wustl.edu/projects/yeast/index.php, [9]) using the spore clone of CBS7001, strain 623-6c *ura3-1* ( = CLIB533, CBS9787). The genome of *S. uvarum* CBS7001 has been recently upgraded by the *Saccharomyces Sensu Stricto* consortium (www.SaccharomycesSensuStricto.org) [10]. Sequence annotations confirmed that the *S. uvarum* genome is exempt of *S. cerevisiae* sequences. In 2005, based on sequence data, *S. uvarum* was proposed to be reinstated as a distinct species, thereby abolishing its synonym status with *S. bayanus* [5]. However, *S. uvarum* genomes are still listed as those of *S. bayanus* in the SGD, Génolevures, and NCBI databases to accommodate its synonymy status. For the same reason, sequences identical to those of *S. uvarum* have been labelled in databases as *S. bayanus* sequences because they were obtained from synonym strains of *S. bayanus*. Very recently, some authors have reminded that those genomes are *S. uvarum* and not *S. bayanus* as classified by the sequencing groups [11].

The taxonomic reinstatement of *S. uvarum* (Beijerinck) [5] as a real species has been admitted by some investigators [12], [13], but is still contested by others [14], [15] who argued that the presence of the subtelomeric Y' sequence is not necessarily indicative of a mosaic genome [16], [17]. While at the same time, hybrids have been described with only one marker belonging to each parent species when found concomitantly in one strain [18], [19].

Another controversial interpretation of the differences between *S. bayanus* and *S. uvarum* has been put forward by Rainieri *et al.* [20]: the *S. bayanus* taxon defined in [4] is a heterogeneous complex composed of pure and mixed genetic lines. Although the authors of this study do agree that the type strain of *S. bayanus* CBS380^T^ represents a mixed line, they designated three other strains to be representatives of the pure line: NBRC1948, NBRC539, and NBRC2031. Isolated from beer, these strains are not known as *S. uvarum*, even though the sequences of some markers display more than 99% nucleotide identity with *S. uvarum* strain CBS7001 (see for example the sequences Acc N°: AB196324-AB196327, AB196329-AB196331).

The way *S. bayanus* and *S. uvarum* have been grouped has thus been the root of conflicting interpretations. Some propose that these species should be recognized as two varieties of the same species. This suggestion was put forth by G. Naumov [15] who relied upon the biological species concept, a taxonomy concept that was first proposed for plants, where varieties are inter-fertile [21]. The concept is partially derived for yeast based upon the notion that full fertility occurs when strains of the same species are crossed, whereas half fertility defines varieties within a species. Yeast genomic studies have led to new insights into the biological species concept since reduced fertility or absence of fertility between strains in a species may result from gross chromosomal polymorphism or translocations. This has been observed in a strain of *S. paradoxus* regarded as *S. cariocanus,* a distinct species [22]. Inter-fertility is thus an all-or-nothing phenomenon. Although appealing, the biological species concept is therefore difficult to apply in many cases. A further complication stems from the fact that interspecies gene transfers described between *Saccharomyces* yeasts [23] appear much more common than previously anticipated, thus blurring species boundaries. This has been largely confirmed by recent genome sequencing data now available for many *Saccharomyces* strains [22], [24], [25], [26] indicating that up to 10% of the strains classified in collections as *S. cerevisiae* may be natural hybrids between *S. cerevisiae* and more or less closely related species [24]. This situation may also prevail for other species such as *S. bayanus*.

To try and better determine the delineation between *S. bayanus* mixed-line, pure-line, and *S. uvarum*, we decided to investigate the genomes of representative strains of the above species using, as references, the genomes of *S. cerevisiae* S288c, EC1118, and *S. uvarum* CBS7001 ( = MCYC623) available in public databases. In a first step, we amplified and sequenced 17 *S. uvarum* genes which revealed that *S. bayanus* strains present a mosaic genome including *S. uvarum* sequences and more divergent “*S. uvarum*-like” sequences. To differentiate hybrids from the genuine species, we initially relied upon physiological characterisation followed by PCR amplification-and-sequencing of specific markers from *S. cerevisiae.* Some of these markers were then localised on electrokaryotypes by chromosomal blotting and Southern hybridisation. In addition, whole genome scanning using Comparative Genomic Hybridization array (aCGH) was performed to detect all possible *S. cerevisiae* materials of suspected mosaic genomes. Beside the *S. bayanus* type strain CBS380^T^, we also analysed the beer strains NBRC539 and NBRC1948 which have been described as non-hybrid, pure genetic lines of *S. bayanus* [20]. As *S. bayanus, S. uvarum* and *S. cerevisiae* have been regarded as contributors to the *S. pastorianus* genome [27], we included *S. pastorianus* CBS1538^NT^, *S. carlsbergensis* CBS1513 and *S. monacensis* CBS1503 into our study. We also included the strain NRRLY-1551, since this strain has been confused with CBS1538^NT^ and been used many times instead of the *S. pastorianus* neo type strain. We revealed three *S. cerevisiae* contigs with as many as 27 genes in strain CBS380^T^ confirming that it indeed carries a mosaic genome. This was also the case for strains NBRC539 and NBRC1948 since two more chromosome contigs of *S. cerevisiae* origins were found in the genome of these strains. Obviously these transfers have resulted in the capacity to efficiently metabolise not only maltose but also maltotriose, two sugars which are abundant in beer wort. We then checked the fertility between *S. bayanus* and *S. uvarum* using strain *S. bayanus* NBRC1948 which sporulates and ensures high spore viability instead of strain CBS380^T^ which was infertile in our hands. The hybrid NBRC1948 and the pure line CBS7001 were crossed and were fully inter-fertile, resulting in F1 spores with chromosomal patterns similar to CBS380^T^ and NRRLY-1551. These strains were thus elements of tetrads issued from crosses involving either the *S. uvarum* and strain NBRC1948 or a similar strain.

We also compared the *MEL*1 gene amplified and sequenced from *S. uvarum*, *S. bayanus* CBS380^T^ and *S. cerevisiae* Mel^+^ strains. This comparison revealed that *S. carlsbergensis MEL*1 gene is different from the *SuMEL*1 gene in *S. uvarum*. The Mel+ character has reduced *S. carlsbergensis* to a synonym of *S. uvarum* [3] making it possible to suggest that *S. carlsbergensis* is the same as *S. uvarum* [28], [29].

Finally, the phylogenies obtained for many of these *cerevisiae* and non*-cerevisiae* markers, combined with microsatellite data on *S. cerevisiae* populations enable us to propose a new phylogeny of these beer lineages.

## Results

### Genome analysis

We investigated the genomes of the *S. bayanus* (Saccardo) strain group (Table 1) regarded as hybrid or pure lines such as CBS380^T^ and NBRC1948 by comparing them with the genomes of the related species *S. uvarum* (Beijerinck) strains CBS395^T^ or CBS7001, as well with the genome of *S. cerevisiae* and *S. pastorianus* lager strains CBS1538^NT^, CBS1513 (ex *S. carlsbergensis*) and CBS1503 (ex *S. monacensis).*

**Table 1:** List of *Saccharomyces* strains.

CBS number	Other numbers	previous names	NTS2 fingerprints	Reclassification	Origines (§), remarks
**380^T^** (s)		*S. bayanus*	CARB		CBS
**395^T^**		*S. bayanus*	UVAR	*S. uvarum*	CBS
7001(g)	MCYC 623	*S. bayanus*	UVAR	*S. uvarum*	CBS; genome labeled *S. bayanus* in SGD
378		*S. bayanus*	UVAR	*S. uvarum* hybrid	CBS
	CLIB 271	*S. uvarum* hybrid	UVAR		CLIB
2946		*S. bayanus*	CARB	*S. uvarum*	CBS
424		*S. globosus*	CARB		CBS
	NBRC 1948	*S. bayanus*	CARB		NITE
	NBRC 539	*S. bayanus*	CARB		NITE
	NBRC 2031	*S. bayanus*	UVAR	*S. uvarum* hybrid	NITE
	NBRC 2003	*S. pastorianus*	SACE		NITE
1513		*S. carlsbergensis*	CARB		CBS
1503		*S. monacensis*	CARB		CBS
**1538^NT^**		*S. pastorianus*	CARB		CBS, Neo type of *S. pastorianus*
	CLIB 276*	*S. pastorianus*	CARB		Beer, MUCL 28282
	CLIB 277*	*S. pastorianus*	CARB		Beer, MUCL 28283
	CLIB 278*	*S. pastorianus*	SACE		Beer, MUCL 28284
	CLIB 279*	*S. pastorianus*	SACE		Beer, MUCL 28285
	NRRL Y-1551	*S. pastorianus*	CARB	*S. bayanus*	Ceased to exist in the ARS collection
	S288c(g)	*S. cerevisiae*	SACE		YGSC
	YNN295	*S. cerevisiae*	SACE		YGSC
2354		*S. cerevisiae*	SACE		CBS
	ATCC 42367	*S.carlsbergensis*	SACE	*S. cerevisiae*	ATCC
5287	CLIB 219 (*ade2*)	*S. cerevisiae*	SACE		Tester strain, Naumov *et al*. [40]

(*)Strains used only in microsatellites analysis.

(g) Genome sequence available,

(s) genome surveyed sequences available.

ATCC American Type Cultures Collection, CBS Centraalbureau voor Schimmelcultures, CLIB Collection de Levures d'Intérêt Biotechnologique, MUCL Microbiology Collection of Yeasts Cultures, NITE ( = NBRC) Biological Resource Center, YGSC Yeast Genetic Stock Center.

### Detection in *S. bayanus* CBS380^T^ of a 20 kb *S. cerevisiae* fragment extending from the *MAL* locus to the telomere of chromosome VII

When testing the fermentation of maltose, the *S. bayanus* strain CBS380^T^ responded strongly and rapidly, whereas the *S. uvarum* strains responded more slowly (Table 2). We hypothesized that the capacity to ferment maltose might reflect the presence of *MAL* genes originating from *S. cerevisiae*. Primers were then designed from the sequence of S288c at the SGD to amplify *S. cerevisiae MAL33, MAL31* and *MAL32* genes. PCR products were obtained from the genomic DNA of *S. bayanus* CBS380^T^ for *MAL31* and *MAL32* (designated *SbMAL31* and *SbMAL32*) but not for *MAL33*. The latter gene was mutated in S288c, and was later amplified with primers designed from the sequence of the *S. cerevisiae* wine yeast EC1118 [24].

**Table 2:** Distribution of *S. cerevisiae* genes in tetrads from the NBRC 1948-CBS 7001 cross.

Markers	Methodology	CBS	NBRC	NBCB-2				NBCB-6				CBS	NRRL
		7001	1948	2a	2b	2c	2d	6a	6b	6c	6d	380^T^	Y-1551
Contig cB	Hybridization	−	cB	cB	−	−	cB	-	cB	−	cB	cB	cB
Contig cA	Hybridization	−	cA	cA	−	cA	−	−	−	cA	cA	cA	cA
Contig cC	Hybridization	−	cC	−	cC	cC	−	cC	−	−	cC	cC	cC
*SuMEL*	PCR or Seq.cing	+	−	−	+	+	−	−	+	−	+	+	+
*HO*	Sequencing	UVA	LG	UVA	UVA	LG	LG	UVA	LG	UVA	LG	LG	LG
*ScBIO2*	PCR	−	+	+	−	−	+	−	+	−	+	+	+
*ScMAL31* specific fragment (a)	PCR	−	+	+	−	−	+	−	+	−	+	+	+
*MTY1* specific fragment (b)	PCR	−	+	+	+	+	−	+	−	+	+	+	+
*ScMAL33*	PCR	−	+	+	+	+	+	+	+	−	+	+	+
*ScBIO2-ScIMA1*	PCR	−	+	+	−	−	+	−	+	−	+	+	+
*ScIMA1-ScMAL33*	PCR	−	+	+	+	+	+	+	+	***	+	+	+
*ScMAL33-(MTY1-ScMAL31)*	PCR	*-*	+	+	+	+	+	+	+	−	+	+	+
*(MTY1-ScMAL31)-ScMAL32*	PCR	−	+	+	+	+	+	+	+	+	+	+	+
*ScMAL32-ScCOS2*	PCR	−	+	+	+	+	+	+	+	+	+	+	+
*ScCOS2-ScSUC4*	PCR	−	+	+	+	+	+	+	+	+	+	+	+
*ScSUC4-ScRTM1*	PCR	−	+	+	− (c)	− (c)	+	+	+	+	+	+	+
Contig cE	PCR	−	+	−	E	−	E	E	−	E	−	+	+
*NTS2* patterns	PCR-RFLP	UVAR	CARB	UVAR	CARB	CARB	UVAR	UVAR	UVAR	CARB	CARB	CARB	CARB
*PMA1*	PCR-RFLP	UVA	LG	UVA	UVA	LG	LG	UVA	LG	UVA	LG	UVA	LG
*MAL* locus	Sequencing	−	MAL31/MTY1	ND	MTY1	ND	MAL31	MTY1	MAL31	MTY1	ND	MAL31/MTY1	MAL31/MTY1
	Melibiose fermentation	+	+	−	+	+	−	−	+	−	+	−	+
	Maltotriose fermentation	−	+	+	+	+	−	+	−	+	+	+	+
	Maltose fermentation	+	+	+	delay	delay	+	−	+	delay	+	+	+
	Sporulation	+	+	+	+	+	+	−	+	+	+	+	−

(^a^) Primers MAL31yF (5′TGAGTGGTTTTAGCGTATTC)/MAL31SpR1 (5′ CAAAACTGTAACTACAATTTGG).

(^b^) Primers MAL31yF ((5′TGAGTGGTTTTAGCGTATTC)/MTYSpR2 (5′CAATAGGAACCTTCTGAG).

(^c^) *RTM* amplifiable but *SUC4* not amplifiable.

*** The junction between the *uvarum* part and the *cerevisiae* part occurred within this fragment for contig cA and gave rise to a PCR fragment shorter than expected. LG: sequence with 93–94% identity with *S. uvarum* (UVA) sequences.

Sequencing revealed that *SbMAL32* was identical to *S. cerevisiae ScMAL32* whereas *SbMAL31* shared only 90% nucleotide identity with *ScMAL31* but 98% identity with *MTY1*, a gene encoding the maltotriose transporter described in *S. carlsbergensis* [30].

**Figure 1:**
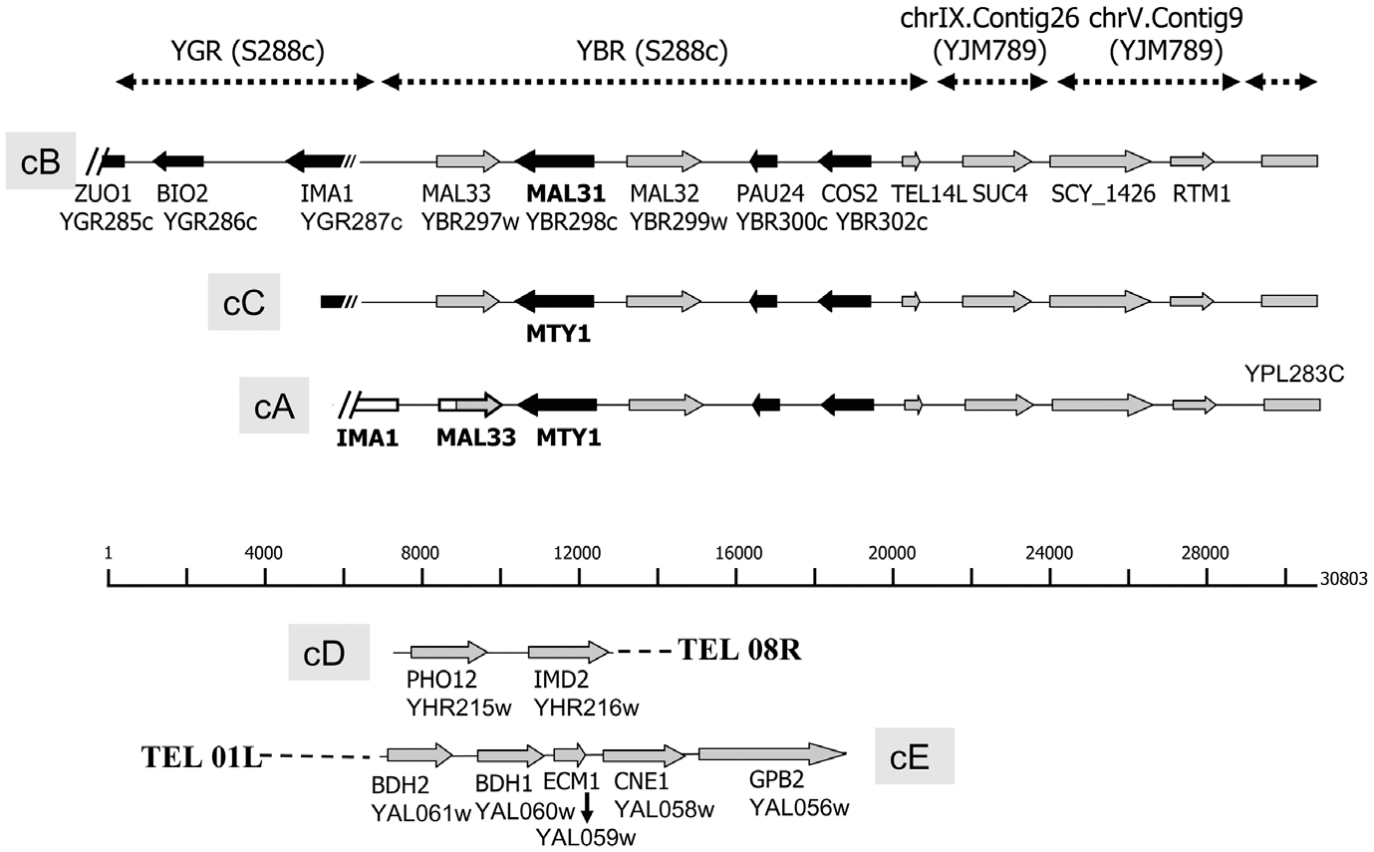
Schematic representation of *S. cerevisiae* contigs in the genomes *S. bayanus* CBS380^T^ and NBRC1948. Black arrows represent to scale genes oriented from the telomere to the centromer and grey arrows genes in the opposite orientation. The junction between *S. uvarum* and *S. cerevisiae* chromosomes was indicated by a colour change (white for *S. uvarum,* grey for *S. cerevisiae*) within the *MAL33* gene of cA. The 5′ ends of contigs A and C were deduced from segregants NBCB-6c and NBCB-6a issued from the cross between strain NBRC1948 and CBS7001.

From the *MAL32* gene in the direction of the telomere, we used chromosome walking to further amplify and sequence *PAU24* and *COS2* on the genomic DNA of CBS380^T^ which were found to be identical to *S. cerevisiae* sequences. In CBS380^T^, all intergenic sequences could be amplified from the regions extending from *MTY1* to *COS2* as well as the *COS2*-telomere region. This last fragment that contain *SUC4* and Y' of *S. cerevisiae* and that has been previously sequenced in CBS380^T^ [5], [6], was assigned to the newly identified region. Finally a contig of 20kb, named SC20, was assembled spanning the following genes: *MTY1*-*MAL32*-*PAU24-COS2-SUC4-SCY_1426-RTM1-*Y' (Figure 1). *RTM1* has been reported to be responsible for the resistance to molasses toxicity in industrial *S. cerevisiae* strains [31]. All intergenes of SC20 shared 99% sequence identity with their counterparts in *S. cerevisiae*. Thus, the entire region, except for the coding sequence of *MTY1*, has originated from *S. cerevisiae* genome.

As the two *S. bayanus* strains, NBRC1948 and NBRC539, have been admitted as genetically pure-lines [20], they should be devoid of the *S. cerevisiae* genes observed in *S. bayanus* CBS380^T^. To verify the purity of these genomes, PCR amplifications with different combinations of primers for the contig SC20 were performed with genomic DNA from NBRC1948 and NBRC539. The fragments *MTY1*_*MAL32,* and *SUC4_RTM1* from NBRC1948 and NBRC539 were identical with the corresponding fragments from CBS380^T^. This suggests that SC20 is also present in NBRC1948 and NBRC539 and that these strains harbour the same composite genomes as *S. bayanus* CBS380^T^. We therefore used both NBRC1948 and CBS380^T^ strains in further experiments.

### Three chromosomes of *S. bayanus* CBS380^T^ and NBRC1948 bear the *S. cerevisiae* contig SC20

**Figure 2:**
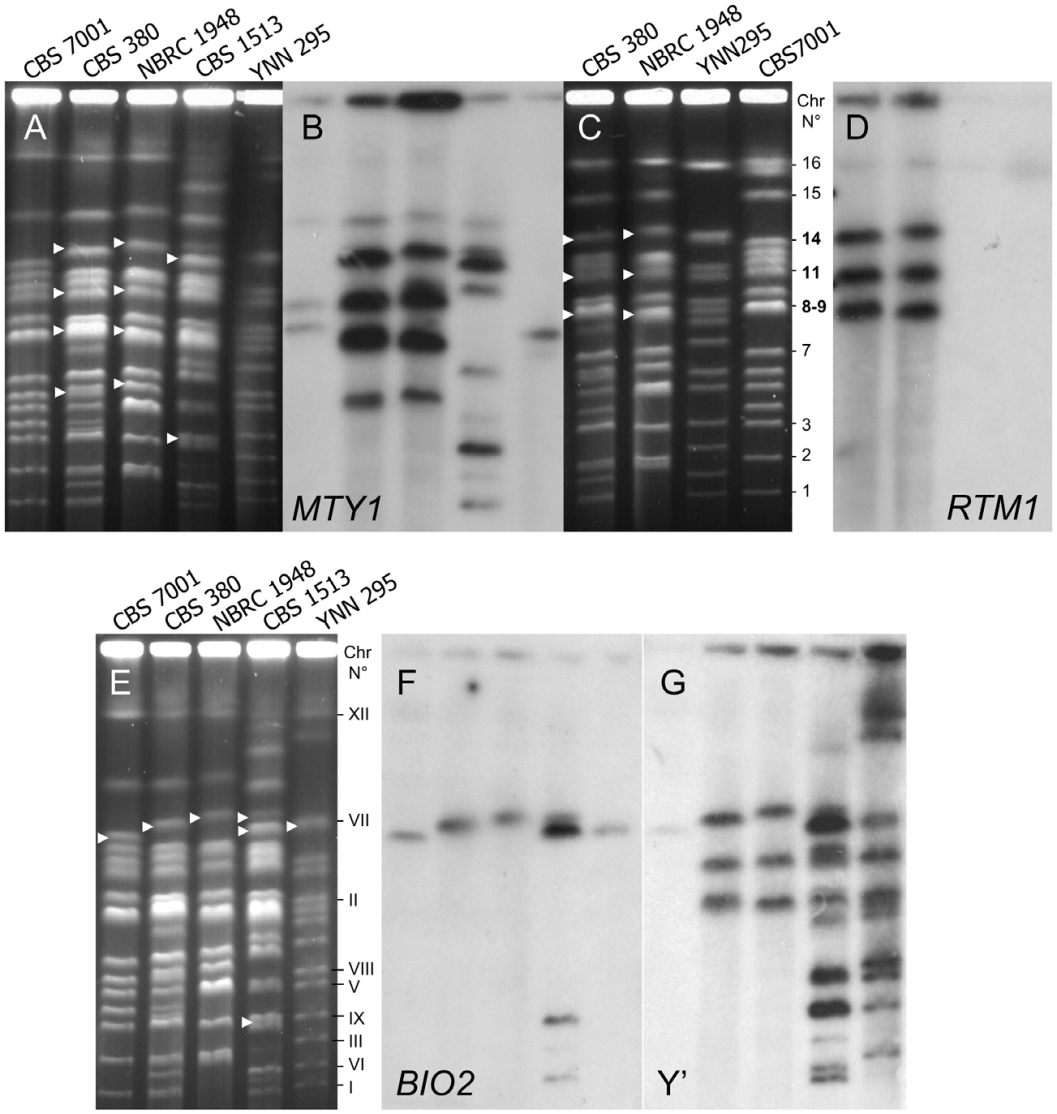
Chromosomal localisation of some *S. cerevisiae* genes in *S. bayanus* strains. A, C, E: Electrophoretic karyotypes of yeast strains stained with ethidium bromide. B, D: Southern blot hybridisation on karyotypes A and C, respectively, with probes *MTY1* and *RTM1* amplified from CBS380^T^. F, G: Southern blot hybridisation on karyotypes (E) for indicated strains with successively *BIO2* and Y' from S288c. Arrow heads indicate chromosomes of *S. bayanus* (CBS380^T^ and NBRC1948) and *S. carlsbergensis* (CBS1513) strains revealed by the probes. *S. uvarum* chromosomes are numbered according to [6].

Localization of markers from contig SC20 on the chromosomes of *S. bayanus* CBS380^T^ and NBRC1948 was performed by karyotype comparison with the *S. uvarum* strain CBS7001 and the *S. cerevisiae* strain YNN 295 (Figure 2A). Chromosomal blotting and Southern hybridisation with *MTY1* and *RTM1* —two markers located at both extremities of the contig SC20— showed that the SC20 contig is repeated on three chromosomes of CBS380^T^ (Figure 2B, 2D) as previously reported for *SUC4* and Y' [6]. The same localisation was also observed for strain NBRC1948 although its karyotype is not identical to that of CBS380^T^. As expected, the Y' probe was also localised in the same chromosomal bands as *RTM1* in strains CBS380^T^ and NBRC1948. *S. cerevisiae* YNN295 does not contains *RTM1* (Figure 2D), whereas no signal of these *S. cerevisiae* markers was obtained on the *S. uvarum* CBS7001 chromosomes as expected. The *MAL31* homologue from *S. uvarum* presents only 77% identity with *S. cerevisiae MAL31*. We designated the three *S. cerevisiae* contigs, cB, cA, and cC, located on the three composite chromosomes of CBS380^T^ and NBRC1948. These three chromosomes have respective sizes which are comparable to those of chromosomes 14, 11, and 8–9 in the strain *S. uvarum* CBS7001 (Figure 2C). Chromosomes are numbered as in [4]. Previously, the *S. cerevisiae MAL* genes have been detected on the chromosomes of strain CBS380^T^ by Southern hybridization [17]. However, in the absence of sequencing, the authors did not recognize the *MTY1* gene.

### Detection of other *S. cerevisiae* fragments in strains CBS380^T^ and NBRC1948

We searched for further *S. cerevisiae* content in strains CBS380^T^ and NBRC1948 using array-CGH mapping. For this, we employed Affymetrix Yeast2 arrays which are specific for *S. cerevisiae* DNA [32]. The strain *S. uvarum* CBS7001 was used as a reference as it is known to be devoid of *S. cerevisiae* genes [7], [9],

### Array-CGH analysis of the CBS380^T^ and NBRC1948 genomes

After CGH scanning, the log-ratio calculated for each probe was plotted along each chromosome. This revealed the presence of several regions with hybridization signals stronger than the *S. uvarum* CBS7001 background, suggesting the presence of *S. cerevisiae* genes. Examples are shown for *S. cerevisiae* chromosomes A, B, and H (Figure S1). Three regions larger than 1 kb with strong hybridization signals were detected in CBS380^T^. These corresponded to YB (*S. cerevisiae* chromosome B) from nt 801475 to nt 809216, YG from nt 1063994 to nt 1067828, and a telomeric region carrying Y' (Table S1). These three regions represent a total of 17.3 kb of *S. cerevisiae* S288c genomic DNA. The first and the third regions include almost all of the genes amplified and previously assembled into contig SC20: *MAL33*, *MAL31* or *MTY1, MAL32,* and *PAU24*, which are located in the subtelomeric regions of *S. cerevisiae.* The second region bears *ZUO1*, *BIO2*, and *IMA1* encoding an isomaltase (α-glucosidase) activity [33].

Compared with CBS380^T^, strain NBRC1948 presented a higher proportion of *S. cerevisiae* genes in its genome with 21 regions >1 kb totaling as much as 89 kb (Table S3). These included the *S. cerevisiae* regions already found in CBS380^T^ and many more *S. cerevisiae* genes —such as *PHO12, IMD2,* and *FLO5* on YHR— as well as the YAL genes *BDH2, BDH1, ECM1, CNE1,* and *GBP2*. Thus in CBS380^T^ and NBRC1948, aCGH detected a *S. uvarum* genetic background interspersed with *S. cerevisiae* fragments or regions.

The classification of all of these regions into GO categories revealed that three categories of genes were overrepresented: genes belonging to the ribonucleoprotein complexes (20 genes), genes involved in key functions under anaerobiosis or high osmotic stress such as *AUS1, PBS2, GPD1, TDH1 GPH1*, and genes belonging to the maltose metabolic process (three genes of the *MAL* locus).

### A *S. cerevisiae* contig cB of 30.8 kb in strains CBS380^T^ and NBRC1948

The aCGH analysis identified *ZUO1*, *BIO2,* and *IMA1* which are contiguously located on the right arm of chromosome VII, YGR of S288c. Using *S. cerevisiae* primers on CBS380^T^ and NBRC1948, we could amplify and sequence a BIO2 block containing a truncated sequence of *ZUO1*, the entire sequence of *BIO2*, and a truncated *IMA1*.

Using Southern blotting, the *BIO2* gene was detected on a single chromosome bearing the contig cB in strains CBS380^T^ and NBRC1948 (Figure 2E, 2F). We hypothesized that cB may start in the *BIO2* block and extend to SC20. Indeed, a 10 kb fragment spanning from *BIO2* to *MTY1* was successfully amplified in these two strains using the Expand Long Range PCR kit. Sequencing of the unknown parts of this 10 kb-fragment allowed us to complete and correct the *MAL* locus with *MAL33-MAL31* (in place of *MTY1)* genes at the 3′ end of the *ZUO1*-*BIO2*-*IMA1* block. In the final assembly, contig cB is 30803 bp in length, spanning the following genes: truncated *ZUO1, BIO2*, truncated *IMA1*, and *MAL33-MAL31-MAL32-PAU24-COS2-TEL14L_XC-SUC4-SCY_1426-RTM1-YPL283C* (Accession number FN677930). The block *SUC4, SCY_1426* and *RTM1*, has been recently described in two brewing yeasts [34].

In the genomes of the *S. bayanus* strains CBS380^T^ and NBRC1948, both *MAL31* and *MTY1* genes exist and respectively encode maltose and maltotriose transporters. Southern blotting showed that the *MTY1* probe hybridized with three chromosomes on the karyotypes of CBS380^T^ and NBRC1948. As *MTY1* and *MAL31* share 90% nucleotide identity, cross hybridization of *MTY1*/*MAL31* was observed. Later, segregants from the cross NBRC1948 x CBS7001, carrying single contig cB, cA, and cC, allowed us to show that cB carries *MAL31*, whereas cA and cC both carry *MTY1* (Figure 1). This was confirmed by sequencing the *MAL* locus in each segregant separately. In either segregants or parent strains, the presence of *MAL31*, *MTY1*, or both can be recognized by PCR using specific primers (Table S2).

### Presence of cB contig in brewing yeasts

*S. carlsbergensis* CBS1513 can ferment maltotriose. Salema-Oom *et al.* [30] attribute this activity to *MTY1* whereas Alves *et al.* [35] have implicated the *AGT1* (or *MAL11*) gene. The *BIO2*_*MAL31/MTY1* fragment was amplified in the *S. pastorianus* strains CBS1513 (ex *S. carlsbergensis)* and CBS1503 (ex *S. monacensis*), but not in the *S. pastorianus* CBS 1538^NT^ (Table 3). In the *S. carlsbergensis* karyotype, the *BIO2* probe hybridized with one chromosome, whereas the *MTY1* probe hybridized well with four other chromosomes, and the Y' probe hybridized with many chromosomes (Figure 2B, 2F, 2G). *SCY_1426* and *RTM1* are also present in CBS1513 and CBS1503, so we concluded that contig cB carrying *MTY1* or *MAL31* is also present in *S. carlsbergensis* and *S. monacensis*. Indeed, both genes could be amplified from strains of these latter species and sequenced using appropriate specific primers. Out of all the strains used in our study, *AGT1* gene could only be amplified in CBS1513 (*S. carlsbergensis*) and CBS1503 (*S. monacensis*). Their sequences have the same T insertion at nucleotide 1183 which generates a stop codon giving a truncated protein of 394 aa whereas the normal protein has 616 residues (Acc N° FR873106-07). This suggests that *AGT1* may be inactive, thus implying that *MTY1* is responsible for the fermentation of maltotriose in CBS1513 and CBS1503 [30], [36], [37].

**Table 3:** *S. cerevisiae* genes transferred in strains of *S. bayanus* and *S. pastorianus.*

Genes	*S. bayanus*NBRC1948	*S. bayanus*NBRC539	*S. bayanus*CBS380^T^	*S. bayanus*NBRC2031	*S. bayanus*CLIB271	*S. pastorianus*NBRC2003	*S. bayanus*NRRLY-1551	*S. pastorianus*CBS1538^NT^	*S. carsbergensis*CBS1513	*S. monacensis*CBS1503
*BIO2*	+	+	+	+	+	+	+	−	+	+
*MAL33*	+	+	+	+	ND	+	ND	ND	ND	+
*MAL31*	+		+	+	+	+	+	ND	−	+
*MTY1*	+	+	+	−	+	−	+	−	+	ND
*MAL32*	+	+	+	ND	+	ND	+	ND	+*	ND
*COS2*	+	ND	+	ND	ND	ND	ND	ND	+	+
*SUC4*	+	ND	+	+	ND	+	+	+	+	+
*SCY_1426*	+	+	+	+	+	−	+	−	+	+
*RTM1*	+	+	+	+	+	+	+	−	+	+
*MEL1*	−	−	*SuMEL1*	ND	*SuMEL1*	−	*SuMEL1*	−	*MEL* **	ND

Sequences with more than 99% identity with S288c, RTM11-1a, YJM789 and EC1118 are considered as *S. cerevisiae*. For *RTM1* the sequence Acc. N°U02618 was used for comparison. Most of the sequences are deposited (see Table S3).

ND: Not determined.

(*)Acc. N° M12601.

(**)Acc. N° M58484

### Additional *S. cerevisiae* genes present in NBRC1948 and NBRC539

Array-CGH detected some additional *S. cerevisiae* genes in NBRC1948 other than those present in CBS380^T^. We amplified and sequenced some of these genes in NBRC1948. A single 3913 bp fragment spanning the two entire *PHO12* and *IMD2* genes could thus be obtained, defining contig cD (Acc. N° FR754543). Five other contiguous genes *BDH2*, *BDH1*, *ECM1*, *CNE1,* and *GBP2* located on chromosome I (YAL genes) could be amplified in two PCR overlapping fragments: *BDH1_ECM1* and *ECM1_GBP2*. Sequences of these two sub-fragments were assembled into a new 8487 bp contig named cE (Acc. N° FR754541). As expected, no amplification with the primers used was obtained for the above genes in CBS380^T^. Partial *FLO5* (YHR211w) could be amplified and sequenced in strain NBRC1948 (Acc. N° FR754545). Genes *BDH2* and *FLO5* were used as probes on chromosomal blots. *BDH2* is localised on chromosome I of *S. cerevisiae* and on one of the two smallest chomosome bands in NBRC1948. In strains CBS380^T^ and *S. uvarum* CBS7001, *BDH2* of *S. cerevisiae* also marked the smallest chromosome, though with less intensity. This is expected for a 20% diverging sequence (Figure S2A, S2B). *FLO5,* which is a member of a multi-gene family, hybridised with two chromosomes in *S. cerevisiae* and with at least two chromosomes in *S. uvarum* CBS7001 and *S. bayanus* CBS380^T^ but with less intensity (Figure S2C, S2D).

Strain NBRC539, which has been described as another *S. bayanus* genetic pure-line [20] was submitted to several experiments carried out with NBRC1948. Results showed that NBRC539 was similar to NBRC1948. Indeed, both strains shared the same karyotype (Figure S3) and several segments of contig cA, *FLO5* (partial) were amplified and sequenced from NBRC539, as were the cD and cE. These sequences were identical to their counterparts in NBRC1948 (FR754544 and FR754542, Acc. numbers in Table S3). NBRC539 and NBRC1948 are thus genetically similar, although only the latter retained the capacity to sporulate.

### Origin of the *S. cerevisiae* fragments characterized in CBS380^T^ and NBRC1948

The recent re-sequencing of many *S. cerevisiae* strains [16], [24] enabled us to compare the *S. cerevisiae* sequences found in CBS380^T^ and NBRC1948 with sequences from strains of various origins including ale strains [34], or the lager strain Weihenstephan 34/70 [25]. A phylogeny constructed for the *BIO2* nucleotide sequence clearly indicates that CBS380^T^ and NBRC1948 have a wine/European origin, and that the sequence is different from the one encountered in ale yeast strains (Figure S4). Similarly, the Sc*BDH2* gene from NBRC1948 also indicates a wine/European origin which is clearly different from the fragments sequenced in the two ale strains as well as in the lager yeast, Weihenstephan 34/70.

### Differentiation of *S. bayanus, S. uvarum* and *S. pastorianus* strains by fermentation of maltotriose and melibiose

*S. bayanus* CBS380^T^ can ferment maltose more quickly and strongly than *S. uvarum* strains. However, the difference between these species was even more conspicuous when maltotriose was used in fermentation tests; only *S. bayanus* can ferment this trisaccharide, *S. uvarum* strains cannot. Strains CBS380^T^, NBRC539 and NBRC1948 all fermented maltotriose. This activity in *S. bayanus* is likely to be mediated by *MTY1* as demonstrated for *S. carlsbergensis* [30], [36]. Fermentation of maltotriose is thus a common character between *S. bayanus* and the group of brewing yeasts.

Melibiose utilisation is a characteristic common to *S. carlsbergensis* and *S. uvarum*. This latter is known as Mel+ and carries the *MEL*1 gene, which is found in the *S. uvarum* genome (contig AACA01000043). Primers were selected to amplify the *MEL*1 in the *S. uvarum* strains CBS395^T^ and CBS2946, *S. bayanus* CBS380^T^, and strain NRRLY-1551. They all share an identical sequence designated *SuMEL*1 (Acc. Numbers FR750556-59). Sequence alignment showed that *SuMEL*1 displays 79% and 94% nucleotide identities with *MEL1* genes from *S. cerevisiae* (Sc*MEL1,* Acc N° M10604) and from *S. carlsbergensis,* respectively (Acc N° M58484) [38]. The Sc*MEL1* gene was also amplified and sequenced in two *S. cerevisiae* strains, ATCC42367 and CBS2354 (Acc. N° FR75054-55). On chromosomal blotting, the *SuMEL1* probe hybridised to chromosome 3 of *S. uvarum* strains CBS395^T^ and CBS7001 as well as to its isomorphic chromosome in *S. bayanus* CBS380^T^ (Figure S5B, S5D). However, in *S. cerevisiae* Mel+ strains, *S. carlsbergensis* CBS1513, and *S. monacensis* CBS1503, the Sc*MEL1* probe hybridised with a larger chromosome (Figure S5B, S5D). *MEL1* could not be amplified in strains NBRC539 and NBRC1948 with either *S. uvarum* or *S. cerevisiae* primers and could not be detected on the karyotype of NBRC1948 (Figure S5). If the gene is indeed absent, this may explain why these two strains cannot ferment melibiose.

### Inter-fertility of *S. bayanus* NBRC1948 and *S. uvarum* CBS7001

To determine whether *S. bayanus* and *S. uvarum* are conspecific according to the biological species concept, we tested the fertility of a hybrid diploid strain.

Strain CBS380^T^ was able to sporulate albeit poorly giving no viable spores from 20 dissected tetrads under normal growth conditions on rich medium YPD. This strain is therefore practically infertile in our hands. However, Ryu *et al.* [39] have obtained a sporal clone from CBS380^T^, strain B19-3c, presenting a karyotype with three missing bands compared with CBS380^T^ [6].

In contrast, strain NBRC1948 sporulated efficiently. After self-sporulation, 19 asci were dissected giving a spore viability of 54%. Karyotypes of segregants from two complete tetrads were indistinguishable from the karyotype of the parental strain NBRC1948 (data not shown). Since the fertility of NBRC1948 was sufficiently high, we crossed this strain with *S. uvarum* CBS7001, which is homothallic and fertile, as self-sporulation and dissection of 24 asci gave 98% of viable spores.

**Figure 3:**
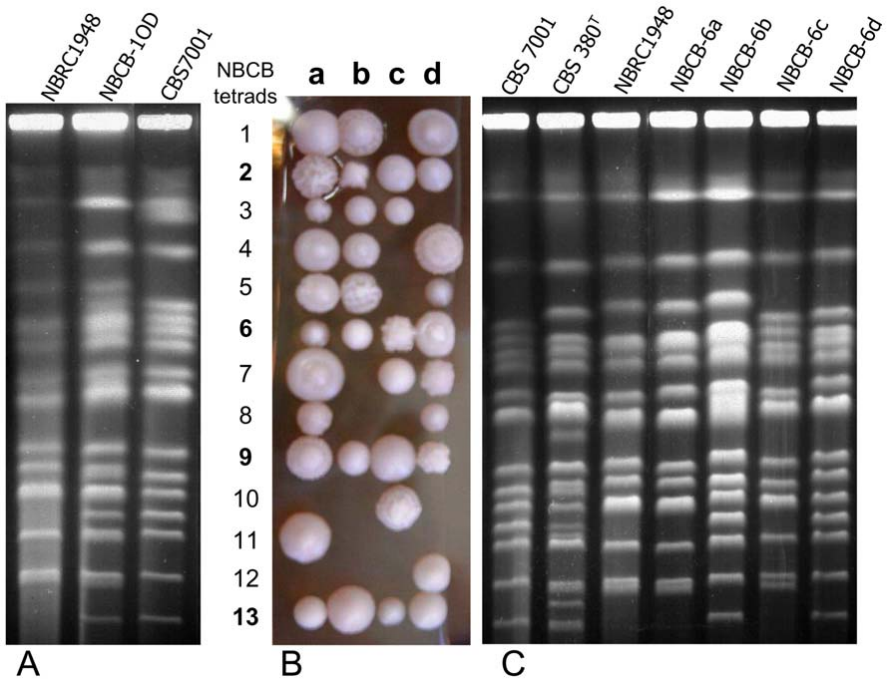
Fertility between *S. bayanus* NBRC1948 and *S. uvarum* CBS7001. A: Karyotypes of the constructed hybrid NBCB-10D carrying two chromosome sets of the parent strains. B: Spore viability in 13 tetrads dissected from this *S. bayanus/S. uvarum* hybrid NBCB-10D. Tetrads are numbered from top to bottom and segregants from left to right. Numbers in bold indicate complete tetrads. C: Electrophoretic karyotypes of the segregants of tetrad NBCB-6 compared with the parent strains NBRC1948 and CBS7001 showing the segregation and recombination of *S. bayanus* and *S. uvarum* chromosomes.

Two hybrids were constructed by crossing strain NBRC1948 with CBS7001 using the spore-to-spore mating technique [40], [41]. One hybrid named NBCB-10D was confirmed by karyotyping and retained for further study. Its karyotype united two sets of chromosomes, each derived from one parent (Figure 3A). As expected, the hybrid cumulated the phenotypes of the two parental strains since it can ferment both maltotriose and melibiose. Thirteen asci were dissected from the sporulated NBCB-10D: 36 spores have germinated giving 69% of viable spore clones. This viability is in the range observed for many intraspecific crosses within *S. uvarum* strains, *i.e.* 45 to 85% [40]. Four complete tetrads, five with three, one with two, and three with one viable spore were obtained (Figure 3B). The four complete tetrads named NBCB-2, NBCB-6, NBCB-9, and NBCB-13 were further analysed; chromosomal patterns of two parent strains and segregants of the tetrads NBCB-6 are shown (Figure 3C). This proved that the cross *S. bayanus* NBRC1948 x *S. uvarum* CBS7001 produced fertile offspring. As a control, an interspecies hybrid was constructed by crossing NBRC1948 with CLIB219 (*ade2*), a *S. cerevisiae* tester strain [40]. The hybrid *S. bayanus* x *S. cerevisiae* was confirmed by the white colour of the colony and its capacity to grow at 37°C was inherited from *S. cerevisiae*; *S. bayanus* cannot grow at this temperature. This hybrid sporulated well and 26 asci were dissected, but no viable spore was obtained on rich medium. Consequently *S. bayanus* is conspecific with *S. uvarum* but not with *S. cerevisiae* according to the biological species concept [42].

### Segregation of *S. cerevisiae* contigs in the NBRC 1948 x CBS 7001 offspring

**Figure 4:**
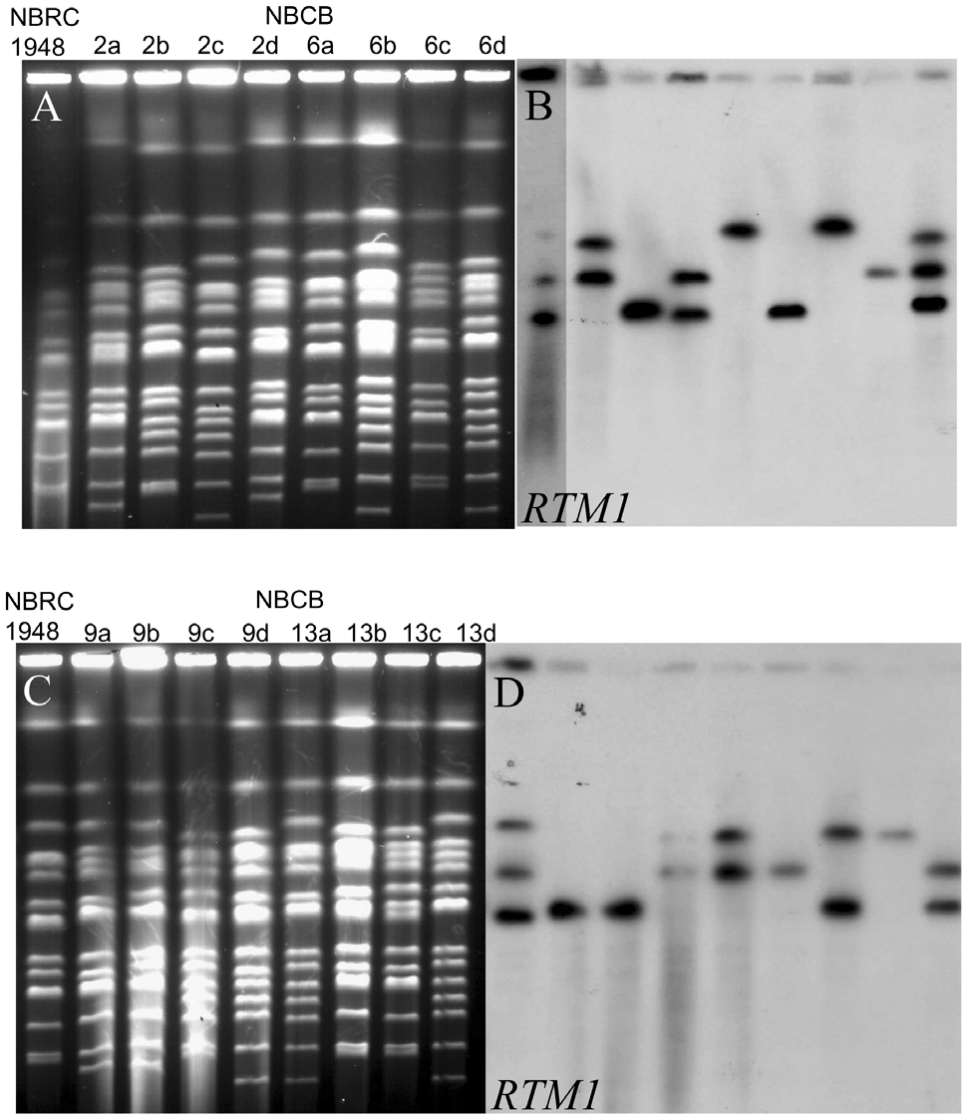
Segregation of *S. cerevisiae* contigs in four tetrads issued from NBCB-10D. A and C: Electrophoretic karyotypes of segregant strains and NBRC1948 parent strain stained with Ethidium bromide. B and D: Southern hybridization with *RTM1* probe amplified from strain CBS380^T^.

The offspring of the cross *S. bayanus*/*S. uvarum* allowed us to follow the transmission of the *cerevisiae* contigs and several other markers in the segregants of the tetrads. Karyotypes of the four complete tetrads from the cross NBRC1948 x CBS7001 showed segregation of the two chromosome sets from both parental strains (Figures 3C, 4A, 4C). In some tetrads (NBCB-2, NBCB-9), none of the karyotypes are completely similar to one parental karyotype and the chromosomes have been reassorted. Chromosomal recombination was clearly observed in segregants NBCB-2a, NBCB-2d, NBCB-9a, and NBCB-9b changing the size of the shortest chromosome which was isomorphic with *S. uvarum* chromosome 1 (Figure 4A, 4C).

Chromosomal blotting following Southern hybridization was first performed with *RTM1* which hybridized with one, two or three chromosomes in the segregants (Figure 4B, 4D). Probing with *MTY1* showed that it also hybridised with the chromosomes bearing *RTM1* in each segregant, whereas the *BIO2* probe hybridised with two segregants bearing the contig cB (data not shown). Thus, the three *S. cerevisiae* contig cA, cB and cC segregated as three independent markers during meiosis. Tetrads NBCB-2 and NBCB-6 were analysed more extensively and we detected segregants bearing single contigs: contig cB in NBCB-2d and NBCB-6b, contig cA in NBCB-6c, and contig cC in NBCB-6a. The segregant NBCB-6d harbours the three contigs cB, cA, and cC. The continuity of the three contigs was confirmed by PCR of segregants NBCB-2d and NBCB-6b (cB), NBCB-6c (cA), and NBCB-6a (cC). Seven overlapping fragments were successfully amplified for contig cB and six overlapping fragments were amplified for cA and cC using a set of suitable primers (Table S2). Sequencing of the *MAL* genes in NBCB-6b, NBCB-6c, and NBCB-6a confirmed the presence of the usual *S. cerevisiae MAL* locus composed of *MAL33*, *MAL31,* and *MAL32*, in contigs cB and the modified *MAL* locus composed of *MAL33*, *MTY1* and *MAL32* in contigs cC and cA (Figure 1). *MTY1* encoding a maltotriose transporter has been first cloned and sequenced in *S. carlsbergensis* but the same gene named *MTT1*, has been later identified in many *S. pastorianus* strains [36]. The sequence of *MTT1* or *MTY1* is divergent but to a lesser degree with *S. cerevisiae MAL31* (90% nucleotide identity) than with the *S. uvarum MAL31* homologue (77% nucleotide identity).

PCR amplification of *ScIMA1-ScMAL33*, yielded a shorter fragment than expected for contig cA in segregant NBCB-6c. Sequencing showed that the *MAL33* gene is a chimer; at the 5′-end, 397 nucleotides have 91% identity with *S. uvarum MAL33* (78% with *S. cerevisiae*) whereas at the 3′-end, there are 1010 nucleotides which are identical to the *S. cerevisiae* strain RM11-1a. Thus, the junction between the *S. uvarum-*like chromosome part and the *S. cerevisiae* contig cA occurred in the *MAL33* gene. The size difference in the segment *ScIMA1-ScMAL33* in cA compared with cB or cC is due to the nucleotide polymorphism of the intergenic region between *S. uvarum-*like *IMA1* and *MAL33* (992 bp in cA sequence Acc. number FR845777 *versus* 2098 bp in contig cB). Both amplicons of *ScIMA1-ScMAL33* were obtained from NBRC1948, CBS380^T^, NRRLY-1551 and from segregants carrying cA and cB (Figure S6A).

PCR amplification using specific primers for *MAL31* and *MTY1* also confirmed the presence of *MAL31* in cB and *MTY1* in cA and cC (Figure S6B). Furthermore, PCR/RFLP of *MAL31/MTY1* with *Hinf*I can be used to recognize strains carrying *S. cerevisiae MAL31* or *MTY1* or both (Figure S6C).

When checked for the capacity to ferment maltose and maltotriose, segregants NBCB-2b, NBCB-2c, and NBCB-6c containing *MTY1* but not *MAL31* fermented maltotriose more rapidly than maltose. NBCB-6a which also possesses *MTY1* but not *MAL31* did not ferment maltose at all. This could be explained by other deficiencies because NBCB-6a grows slowly even on rich medium. Strains NBCB-2d and NBCB-6b carrying *MAL31* but not *MTY1* could only ferment maltose, as expected. Contig cE segregated 2∶2 in the tetrads NBCB-2 and NBCB-6 as confirmed by PCR of the two overlapping regions *BDH2_ECM1* and *ECM1_GBP2* (Table 2). It was localised on one of the smallest chromosome in strain NBRC1948 using the *BDH2* probe (Figure S2B). Other markers non-*cerevisiae SuMEL1* and *HO* segregated 2∶2 as expected for a single gene (Table 2). Segregation of *HO* and *SuMEL1* in the tetrads NBCB-2 and NBCB-6 implies a cross involving two haploid parent strains.

For all of the *cerevisiae* and non-*cerevisiae* makers analysed, the genetic exchanges between *S. bayanus* and *S. uvarum* confirmed that they are fully inter-fertile and the transmission of *cerevisiae* characters —such as the capacity to ferment maltose/maltotriose— to future generations can be assumed by many segregants of this cross.

### Distribution of *S. uvarum* genes and lager-type sequences in *S. bayanus* strains

Our previous results [6] and array CGH data obtained in this study demonstrate that several strains classified as *S. bayanus* carry *S. cerevisiae*-like sequences in a CBS7001-like genomic background (hereafter referred to as *S. uvarum*). Other studies suggest the presence of a third ancestral parent in *S. bayanus* [20], [43], [44], [45]. To confirm the presence of different backgrounds in *S. bayanus* and *S. uvarum* strains, we compared several non-*cerevisiae* sequences of CBS380^T^ with those from different *S. bayanus* and *S. uvarum* strains using primers designed from the CBS7001 genome (Table 4).

**Table 4:** Sequence comparison of *S. uvarum* CBS 7001 genes with homologues of different strains of *S. bayanus, S. uvarum* and *S. pastorianus* (% nucleotide identity).

Genes	Size(bp)	*S. bayanus*CBS 380^T^	*S. uvarum*CBS 395^T^	*S. uvarum*CBS 2946	*S. bayanus*NBRC 1948	*S. carsbergensis*CBS 1513	*S. monacensis*CBS 1503	*S. uvarum*CBS 424	*S*. *bayanus*NRRLY-1551	HybridCLIB 271
**CBS 380^T^ genes identical to ** ***S. uvarum*** ** CBS 7001 homologues**										
*ADE2*	1716	100	100	100	NA	NA	NA	-	NA	100
*ADH1*	1047	100	98.9 (11 sub)	−	100	−	−	−	100	−
*ARG4*	1392	100	100	−	NA	100	−	−	NA	100
*EXG1*	1347	100	100	−	−	NA	93 Lg	−	−	−
*GPI13*	3054	100	99 (9 sub)	99 (1subs)	93 Lg	93 Lg	100	−	−	−
*MEL1*	1416	100	100	100	absent	NA; *ScMEL1*	NA; *ScMEL1*	−	98 (1396/1416)	−
*MET2*	1461	100	100	100	100 partial (1191 bp)	93 Lg	93 Lg	100	100	93 Lg
*NAM2*	2685	100	ND	100	100	−	−	−	−	−
*PGU1*	1086	100	99.9 (1 sub)	100	absent	NA	NA	−	98 (1068/1086)	−
*PMA1*	2751	100	100	100	93 Lg	93 Lg	93 Lg	100	93 Lg	100
*POL1*	4413*	100	100	−	NA	NA; *ScPOL1*	NA; *ScPOL1*	−	−	−
*POL3*	3294	100	100	100	NA	NA	NA	−	−	−
**CBS 380^T^ genes with a majority of neutral SNPs compared with ** ***S. uvarum*** ** CBS 7001 homologues**										
*BAP2*	1830	91 **Lg**	100	−	−	93 **Lg**	93 partial(946 bp)	−	91 Lg	−
*BGL2*	942	93 (882/943)	99.9 (1 sub)	−	NA	NA*; ScBGL2* 940/942	93 Lg	−	−	−
*ERG10*	1197	94 Lg (1134/1197)	100	100	100	94 Lg	94 Lg	100	Mix	Mix
*GDH1*	1365	93	100	100	93	93 ( = Lg *+*1 sub)	93 ( = Lg *+* 1 sub)	−	−	−
*HO*	1761	95 **Lg AB51**	100	99	95	95 **Lg AB50**	−	95	95	99
NTS2 of the IGS (rRNA) *PCR/RFLP*	1244	CARB	UVAR	CARB	CARB	CARB	CARB	CARB	CARB	UVAR

**NA**: absence of amplicon with *S. uvarum* primers; **-**: not determined; **Lg**: described as lager genes by Tamai *et al.*, 2000 [43] or Kodama *et al*., 2001 [44]. By extension, the sequences different from *S. uvarum* and *S. cerevisiae* were also considered as Lg ; **Mix**: multiple amplified fragments, not sequenced; **sub**: nucleotide substitutions between CBS 395^T^ and CBS 7001; **absent**: absence of amplicon with *S. uvarum* or *S. cerevisiae* primers and absence of hybridization bands on Southern blot; ***ScMEL1, ScPOL1***: genes identical to *S. cerevisiae* homologues; **partial**: gene partially sequenced, the size is given within brackets; *POL1 was partially sequenced on 1776 bp. **Lg AB50-51** deposited sequences AB027450, AB027451.

Among the 17 protein-coding genes from *S. bayanus* CBS380^T^, 12 are identical to those of CBS7001 whereas five share around 93% nucleotide identity with their *S. uvarum* counterparts (Table 4). Most of the nucleotide substitutions are neutral so that the deduced protein sequences have over 99% similarity. Four of these five genes share between 99.9 and 100% nucleotide identity with their homologs in *S. carlsbergensis* CBS1513 or *S. monacensis* CBS1503 (Acc. numbers in Table S3; [5]). This type of sequence, which represented 30% (5/17) of the non-*cerevisiae* sequences found in the *S. bayanus* genome, has already been identified in *S. bayanus* CBS380^T^ and NBRC1948 and published as lager-type sequences (Lg sequences [43], [44], [45]). A small set of random fragments of genomic DNA from CBS 380^T^ have been previously compared with *S. cerevisiae* proteins [46]. We revisited these sequences by Blast against contigs of *S. uvarum* CBS 7001 deposited by the MIT and the Washington University (accession numbers AACA00000000 and AACG00000000). Sequences containing protein-coding genes with over 98% nucleotide identity with CBS 7001 genes were considered as being derived from *S. uvarum*. However, this ancestor represented only 40% of the sequences (34 GSSs out of 86, Table S4). Given the quality of the CBS380^T^ sequences (single-strand sequencing) these GSSs cannot be as representative as the sequences obtained in our study. As we found in CBS380^T^ and NBRC1948, non-*cerevisiae* gene sequences are either identical to *S. uvarum* genes (*ADH1, MET2, NAM2,* and *ERG10*) or to lager-type genes (*BAP2, GDH1, GPI13, HO,* and *PMA1)*; we concluded that CBS380^T^ and NBRC1948 carry three types of sequences originating from *S. cerevisiae, S. uvarum*, and a third parent common to *S. carlsbergensis* and other *S. pastorianus* or lager strains.

**Figure 5:**
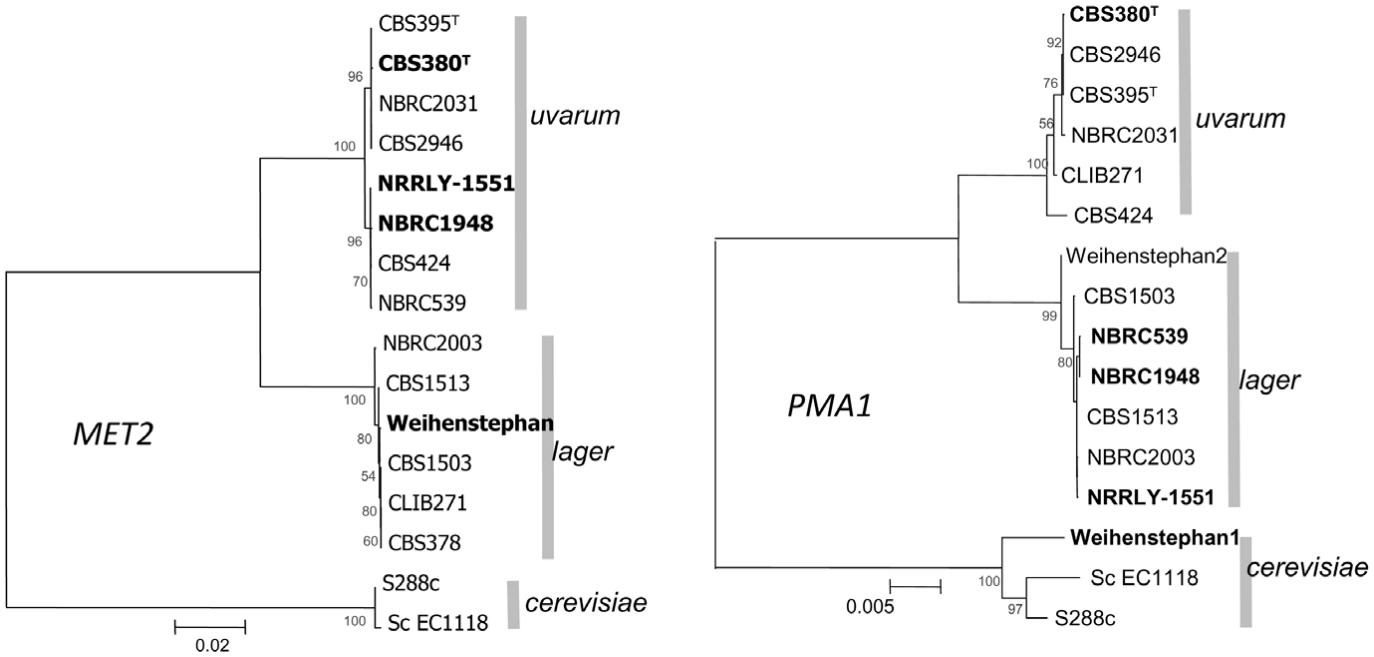
Evolutionary relationships of *MET2* and *PMA1* in mixed and pure lines. The evolutionary history was inferred using the Neighbor-Joining method [55]. The optimal trees are shown (*MET2*: sum of branch length  =  0.24619612; *PMA1*: sum of branch length  =  0.09325799). Bootstrap values calculated on 100 replicates are shown next to the branches. A total of 1529 and 2847 nucleotides for *MET2* and *PMA1*, respectively, were used in the final dataset. Two clusters are clearly separated showing their different origins. This underlines the hybrid nature of some *S. bayanus* strains: NRRL-Y1551, NBRC1948, NBRC539 and *S. pastorianus* Weihenstephan. *S. cerevisiae* sequences were used as outgroups.

Phylogenetic trees based on *MET2, PMA1*, and *MAL31/MTY1* exemplified the variability in strain clustering in *uvarum, lager,* and *cerevisiae* depending on the sequences considered. For instance, strain CBS380^T^ clusters with *uvarum* type for both *MET2* and *PMA1* whereas strain NBRC1948 belongs to the *uvarum* cluster for *MET2* but to the *lager* cluster for *PMA1* (Figure 5).

**Figure 6:**
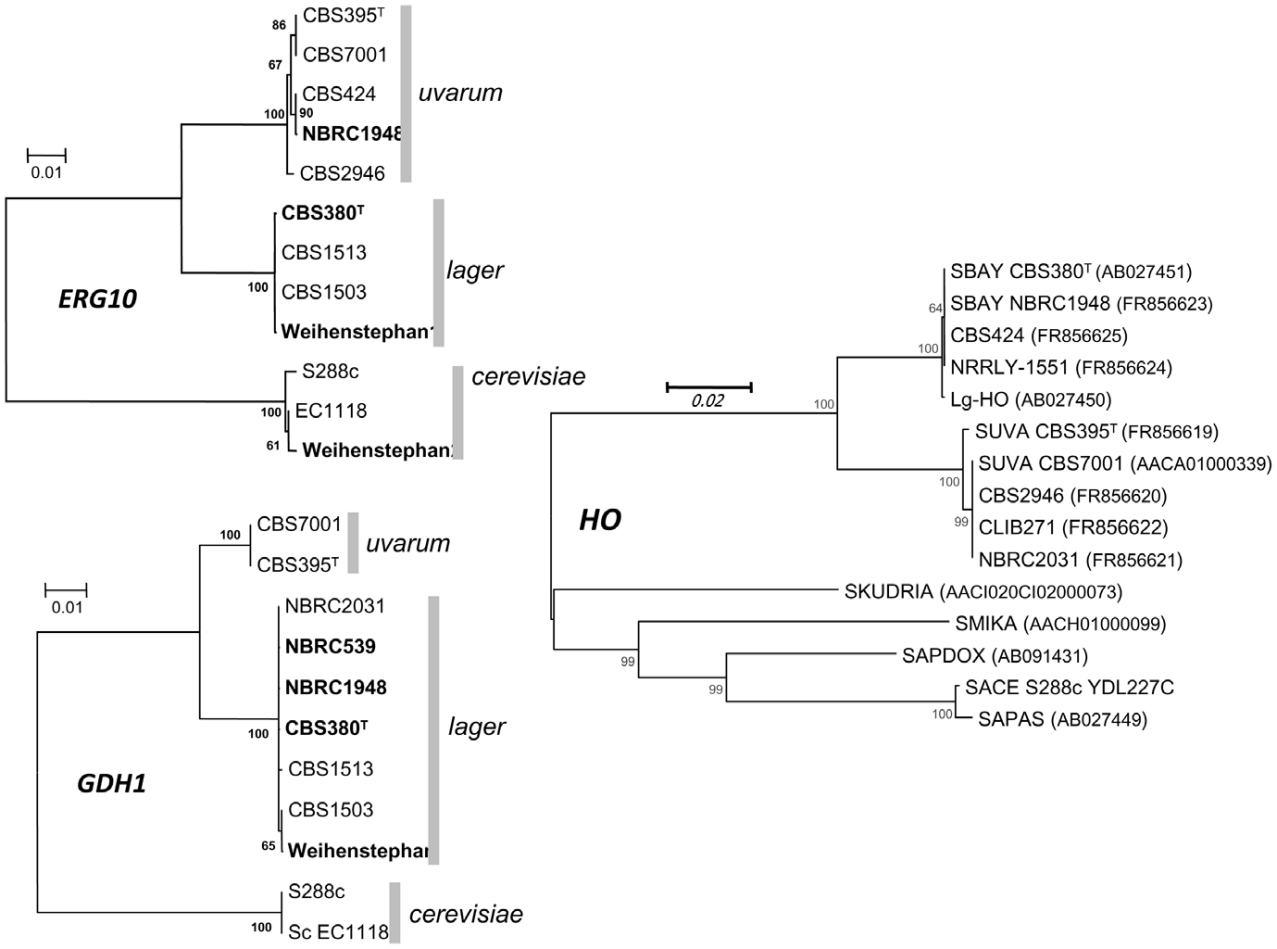
Evolutionary relationships of *GDH1*, *ERG10* and *HO* in mixed and pure lines. The evolutionary history was inferred as in Figure 5 but with limited number of strains analysed for *GDH1, ERG10 and HO*. Sequences *GDH1* have been obtained in this study and from [5], *ERG10* and *HO* are sequenced in this study except for *bayanus*-*HO* and *Lg-HO*. The optimal tree with the sum of branch length  =  0.06421927 is shown. The percentages of replicate trees in which the associated taxa clustered together in the bootstrap test (100 replicates) are shown next to the branches. The tree is drawn to scale, with branch lengths in the same units as those of the evolutionary distances used to infer the phylogenetic tree. The evolutionary distances were computed using the Kimura 2-parameter method [61] and are in the units of the number of base substitutions per site. The analysis involved 44 nucleotide sequences. All ambiguous positions were removed for each sequence pair. There were a total of 4382 positions in the final dataset. Strain CBS 380^T^ clustered in *lager* group based on *ERG10* and *GDH1* phylogenetic trees.

**Figure 7:**
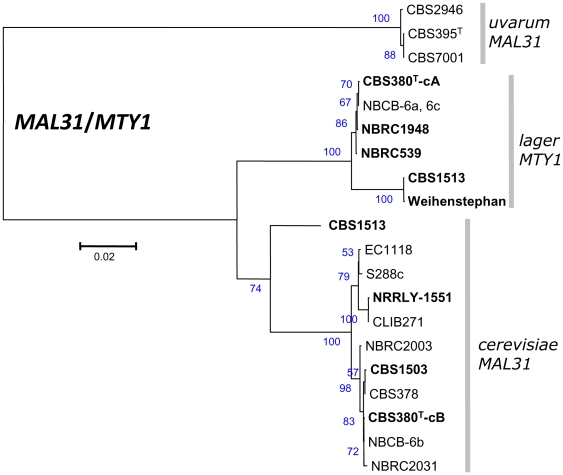
Evolutionary relationships of *MAL31/MTY* in mixed and pure lines. Phylogenetic tree established using the same method as in Figure 6. *MAL3/MTY11* evolution in strain CBS 380^T^, sequence extracted from cB of the segregant NBCB-6b and *MTY1* in cA of the segregant NBCB-6a are considered. In strain NRRLY-1551 only *MAL31* allele could be sequenced.

The *ERG10*, *GDH1* and *HO* sequences were also considered. The trees obtained from these depicted another set of relatedness features: CBS380^T^ is clustered in *lager* whereas NBRC1948 is clustered in *uvarum* for *ERG10* and in *lager* for *GDH1* (Figure 6). The *HO* phylogenetic tree showed two clusters: *bayanus/lager* and *uvarum* (Figure 6). As expected, CBS380^T^ and NBRC1948 clustered with both *lager* and *cerevisiae* regarding *MTY1* and *MAL31* markers (Figure 7). *S. carlsbergensis* CBS1513 with all *lager*-type sequences always belongs to the *lager* cluster reflecting a more *lager*-type than *uvarum*-type background as observed in Table 4. Additional strains were included in the trees and show variable proportions of each background. Based on the three markers *MET2*, *PMA1,* and *MAL31,* NBRC539 and NBRC2031 appear as mixed-line (Figures 5, 7).

### Origin of the *S. cerevisiae* moiety of the genome of lager hybrids

To characterize the origin of the *S. cerevisiae* partner of beer hybrids, the 12 microsatellite loci formerly used to characterize *S. cerevisiae* diversity [47] were amplified in seven beer hybrids: CBS1513, CBS1503, CBS1538^NT^, CLIB276, CLIB277, CLIB278, and CLIB279. Amplification was successful for 5 to 11 loci depending on the strain. Comparison of these allelic profiles with those obtained for different strains by Liti *et al*. [16] and with samples from our database revealed some original features (Figure S7). The different beer isolates were split into three clusters. The first and largest cluster included ale beer strains and lager beer strains of German origin, as previously described [47]. A second group of strains contained *S. monacensis* CBS1503, the neotype strain of *S. pastorianus* CBS1538^NT^, and two other beer strains CLIB276 and CLIB277. Strains of this group exhibit a lower *S. cerevisiae* content in their genomes as only five or six loci could have been amplified. This confirmed the separation of lager yeast into “Frohberg-type” strain and “Saaz-type” strains in agreement with the results of Dunn and Sherlock [45].

Strain *S. carlsbergensi*s CBS1513 clustered away from these two former groups and was more related to some rum and distillery isolates (identical alleles at five loci over 10). These different clusterings indicate that several hybridization events have given rise to these different brewing strains.

## Discussion

Our results present a new picture of beer strain lineages. We show that despite the use of classical or molecular approaches, the hybrid nature of many strains of the *S. bayanus* taxon has until now remained unidentified. Here, we have decisively confirmed that the type strain of *S. bayanus,* CBS380^T^, shows a hybrid nature. Unexpectedly, other strains regarded hitherto as pure genetic lines [20] were also revealed to be mixed genetic-lines: NBRC1948, NBRC539, and NBRC2031.

The hybrid nature of CBS380^T^ has been proposed based on the presence of *S. cerevisiae SUC4,* and Y' in its genome [5], [6]. However, with few *cerevisiae*-genes detected, our proposal has not been generally accepted. New investigations revealing the capacity of CBS380^T^ to strongly ferment maltose have paved the way to detecting more *S. cerevisiae* genes by PCR. Further investigation by aCGH mapping of the CBS380^T^ genome allowed us to amplify and assemble three contigs cA, cB and cC giving a total of 70 kb of *S*. *cerevisiae* sequences. Concerning strain NBRC1948, its hybrid nature has never been suspected even when using PCR/RFLP of three markers per chromosome [20]. This is probably due to the sporadic distribution of *S. cerevisiae* genes in NBRC1948. Here, it was revealed to be a hybrid carrying over 82 kb of *S. cerevisiae* sequences composed of all the *S. cerevisiae* genes found in CBS380^T^ together with contigs cD and cE. This shows that chromosome blotting and Southern hybridization, as well as aCGH, when used separately, may lead to mis-identification of related sequences. For instance, aCGH did not distinguish *MAL31* and *MTY1* in *S. carlsbergensis* [45] because they share 90% identity [30].

### A second species related to *S. uvarum* in the genetic background of *S. bayanus* CBS380^T^ and NBRC1948

As previously reported, our study confirmed the presence of two ancestral partners in the genomes of *S. bayanus* and lager brewing strains [20], [45]. Analysis of the non-*cerevisiae* background in *S. bayanus* NBRC1948, CBS380^T^ enabled us to detect more sequences sharing around 93% identity with their *S. uvarum* counterparts. Some of these alleles have already been described for *S. pastorianus* CBS1503 and CBS1513 and were qualified as *lager*-type [43], [44]. In the present study, additional lager-type markers, such as *BGL2*, *ERG10*, and *PMA1* were amplified with primers designed from *S. uvarum* homologues and sequenced (Table 4). None of these *lager*-sequences were found in the *S. uvarum* genome. In our analyses, the proportion of *lager/uvarum* sequences is 1/3 in *S. bayanus* CBS380^T^ but it seems to be higher in strain NBRC1948 (Table 4) and in *S. pastorianus*.

Strikingly, these *lager*-sequences present a very low nucleotide divergence compared with *S. uvarum* sequences. The SNPs are almost all neutral, which indicates a common origin for both kinds of sequences.

**Figure 8:**
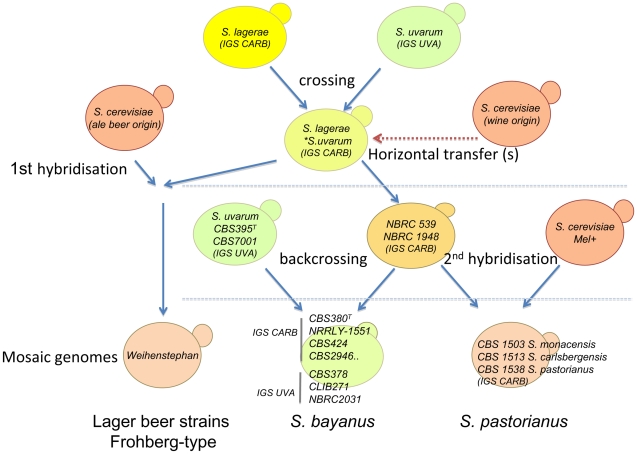
General scheme deciphering the history of crosses between *S. uvarum*, *S. lagerae* and *S. cerevisiae*. Current *S. bayanus* strains are segregants of tetrads issued from a cross *S. uvarum* x NBRC1948 or a similar strain. Strain NBRC1948 represents a generation hybrid anterior to CBS380^T^. *S. lagerae* is the missing species contributor to the genome of strain NBRC1948 or one similar strain. *S. lagerae* materials reflected by Lg sequences that have been transmitted to *S. bayanus* and *S. pastorianus* (Lg presenting 2 to 6% of divergence with *S. uvarum* sequences).

The species carrying these *lager*-type sequences should be the partner of *S. uvarum* forming the genetic background of the *S. bayanus* mosaic genomes. The genome of this *S. uvarum*-like species should present approximately 93% nucleotide identity with *S. uvarum* and be inter-fertile with it. We propose to tentatively name this *S. uvarum* lineage *Saccharomyces lagerae*. Traces of this lineage can be found in strains of the *S. bayanus* taxon such as CBS424 (*e.g*. *S. globosus*) and CBS2946. These strains carry the *S. uvarum* protein-coding sequences analysed but with a CARB type IGS typical of lager strains (Acc. N°AJ243214) as evidenced by the *Alu*I pattern of the NTS2 (Figure S8A) described in [6]. The differential distribution of *lager*-type markers in the genome of CBS380^T^ and NBRC1948 sustains the possibility of independent crosses between each ancestor of these strains. Strains CBS424 and CBS2946 may be considered as mosaic genomes *S. uvarum/S. lagerae*. In addition, strains CBS378, CLIB271, and NBRC2031 without any *lagerae* markers detected might have arisen from a parent strain similar to NBCB-2d or NBCB-6b which was backcrossed with the *S. uvarum* strain. They carry the *S. uvarum* NTS2 and *HO* (Figure S8B) with the usual *MAL* locus carrying *MAL31* but not *MTY1* in two chromosomes of CBS378 [17] and NBRC2031 (data not shown). Another explanation for strains CBS424, CBS2946, CBS378, CLIB271, and NBRC2031 was that they were produced from backcrosses between segregants of NBRC1948 x CBS7001 with *S. uvarum*. This hypothesis is schematically presented in Figure 8.

In traditional brewing conditions, back crossing of NBRC1948 or a similar strain with a *S. uvarum* or with a *S. lagerae* strain might have generated many hybrids which persisted in brewing processes until pure cultures came into practice following the recommendation of E.C. Hansen. Since then, only hybrids capable of fermenting at low temperatures with improved biotechnological characteristics have been selected and maintained. Therefore, the pure *S. lagerae* line endowed with a weak *MAL* system may have been lost in the brewing environment.

### *S. bayanus* consists of two lineages resulting from separated ancestral crosses between *S. uvarum* and *S. cerevisiae*

Our combined molecular analyses revealed that the genome of NBRC1948 was also mosaic with five chromosome contigs of *S. cerevisiae* origin; three of them had been transferred to *S. bayanus* CBS380^T^. In the mosaic genome of NBRC1948, the presence of long *S. cerevisiae* contigs seemed to be the result of transfer events involving the subtelomeric region of three chromosomes. The circulating mechanism that has led to the integration of *Zygosaccharomyces bailii* material into wine yeast genomes [34], [48] is inadequate to explain the presence of contigs cB, cA and cC. This is because these contigs do not show a circular permutation of gene order of the inserted fragment. In addition, the 5′-end junction is different in the three contigs. This suggests that they propagated by unequal crossing over between homologous sequences at subtelomers leading to reciprocal unbalanced chromosomal ends in meiotic segregants. In contig cA, the junction point occurred within the *MAL33* gene which became a chimer with one third *S. lagerae MAL33* and two thirds *S. cerevisiae MAL33*. This corroborates the results reported for strains CBS378 which could be re-interpreted as follows: this strain bears three copies of the *MAL* locus with different truncations in the 5′ ends, one downstream of *MAL33* and another one downstream of *MAL32* [17].

In strain NBRC1948, additional *S. cerevisiae* genes were detected, which constituted a high proportion and greater diversity of genes transferred from *S. cerevisiae.* These findings suggest that NBRC1948 may have arisen from a hybrid *S. uvarum* or *S. uvarum-*like strain which received *S. cerevisiae* DNA fragments in many successive horizontal transfer events. Recurrent backcrosses between this ancestral hybrid and *S. uvarum* strains may have occurred, explaining the progressive loss of *S. cerevisiae* material in some strains isolated from ancient beer. This is exemplified by the results obtained for the NBRC1948/CBS7001 cross. Indeed, karyotypes of tetrads from the hybrid NBCB-10D showed that segregants harbour parental and recombinant chromosomes. However, none of the karyotypes is completely similar to one parental karyotype, as the chromosomes have been reassorted. The segregant NBCB-6d, retains the three *S. cerevisiae* contigs cB, cA and cC, and is very similar to both CBS380^T^ and NRRL Y-1551 except that the latter carries the contig cE whereas CBS380^T^ and NBCB-6d have not inherited it. Based on these features, we propose that strain NBRC1948 is an initial hybrid while CBS380^T^ and NRRLY-1551 are segregants of tetrads deriving from a backcross between NBRC1948 —or a similar strain— with *S. uvarum.*

### Acquisition of new functions by interspecies genes transfer

The *S. cerevisiae* moiety of the mosaic genome of NBRC1948 includes genes with key functions in anaerobiosis (*AUS1*), high osmotic stress (*PBS2, GPD1, TDH1* and *GPH1*), maltose and maltotriose fermentations (*MAL* loci), biotin synthesis, sucrose degradation (*SUC4*), and resistance to inhibitory substance in molasses (*RTM1*). These genes were transferred from a *S. cerevisiae* wine strain, as shown by the phylogenetic trees established with *BIO2* and *BDH2* coding sequences. Thus, the presence of contigs cB, cA, and cC conferred great advantages under brewing conditions with one authentic *MAL* locus (contig cB) and two *MAL*-like loci with *MTY1* (contigs cA and cC) leading to the fermentation of maltotriose. The presence of many genes involved in maltose or maltotriose metabolism in a brewing strain is rather common. Despite the lack of conclusive arguments, the most plausible hypothesis concerning the presence of both *MTY1* and *MAL31* in *S. bayanus* is that two successive events occurred, firstly a transfer of the *S. cerevisiae MAL* locus containing *MAL31* and then the replacement of *MAL31* by *MTY1* by conversion. The biological origin of this latter gene is yet to be determined [37].

Analysis of the offspring of the cross NBRC1948 x CBS7001 clearly showed the independent segregation of the three *S. cerevisiae* chromosomal contigs bearing the *MAL* locus. However, segregants bearing more than one *MAL* locus harbouring two copies of *MTY1* and one copy of *MAL31* would have been more frequently retained. This is because these genes allow these strains to maintain and even increase their capacity to ferment maltose and maltotriose, two saccharides abundant in beer wort. This may explain why different offspring of NBRC1948 carrying the three contigs have persisted until now: CBS380^T^, and NRRLY-1551. These strains resulted from multiple hybridization events to acquire the ability to ferment malt wort at low temperatures as required for lager beer production.

The three genes *SUC4*, SCY_1426, and *RTM1* present in *S. bayanus*, *S. carlsbergensis,* and *S. monacensis* lager yeasts have also been found in the recently sequenced genomes of ale strains [34]. By relying on the genome of *S. cerevisiae* strain S288c, genes present only in wild, or industrial strains such as SCY_1426 and *RTM1* have not been detected in *S. carlsbergensis* and *S. monacensis* [45].

### New proposal for the affiliation of *S. pastorianus*

As proposed by Vaughan-Martini and Kurtzman [4] based on the determination of DNA relatedness between type strains, *S. pastorianus* is regarded as a *S. bayanus*/*S. cerevisiae* hybrid. Since then, studies based on single-locus techniques have produced divergent conclusions. Using the *MET2* gene sequence comparison, *S. monacensis* has been proposed to be the progenitor of *S. carlsbergensis* [49]. However, comparative analysis of the proteomes of CBS380^T^, NRRLY-1551, and *S. pastorianus* obtained by 2D-gel electrophoresis suggested that the proteomes of *S. monacensis* (CBS1503) and *S. carlsbergensis* (CBS1513) result from the superimposition of two patterns. One of these corresponded to *S. cerevisiae* and the other corresponded to strain NRRLY-1551 [50]. In the present study, *MTY1* and the *S. cerevisiae* sequences obtained for *S. pastorianus* CBS1513 and CBS1503 as well as for NBRC1948 and NRRLY-1551 confirmed the relatedness depicted by proteome analysis. Strain NRRLY-1551 is very similar to the F1 segregant NBCB-6d, suggesting that this strain is derived from a backcross between a strain closely related to NBRC1948 and a *S. uvarum* strain. We have indeed shown that some segregants issued from such crosses are fertile (Table 2), and such offspring may then have subsequently mated with *S. cerevisiae* strains to give *S. carlsbergensis* and *S. monacensis*. By analysing the genome of a group of *S. pastorianus* strains using aCGH, Dunn and Sherlock [45] confirmed the *S. uvarum* and *S. uvarum*-like genetic background of lager strains and defined one ale strain as the *S. cerevisiae* partner. Our findings corroborate the triple hybrid nature of these lager yeasts.

### Overall phylogeny of the *S. bayanus/pastorianus* group: diversity of lager yeasts and clarification of the neo type status of CBS1538

We propose a scheme (Figure 8) to resume the generation of strains carrying mosaic genomes from NBRC1948 to CBS380^T^, NRRLY-1551 and other strains in the *S. bayanus* taxon as well as *S. pastorianus* lineages. The two main groups of lager brewing strains are composed of the Froherg-type lager strains including strain Weihenstephan 34/70 and the Saaz-type lager strains including *S. monacensis* CBS1503. These two groups are clearly related to lineages with different *S. cerevisiae*, *S. uvarum*, and *S. lagerae* contents, generating the diversity of strains found in the *S. pastorianus* taxon defined by Vaughan-Martini and Kurtzman [4]. For example many genes of the contig cB could be amplified and sequenced in *S. carlsbergensis* CBS1513 and *S. monacensis* CBS1503 but not in the *S. pastorianus* CBS1538^NT^. Physiologically, the latter cannot utilise maltotriose and melibiose (Mel-), whereas *S. carbergensis* can better utilise these two sugars than *S. monacensis* (data not shown).

Some discrepancies found in the literature on *S. pastorianus* may be attributed to the misuse of two of its neotype strains: CBS1538^NT^ (from the CBS) and NRRLY-1551 (from the ARS in the past). In the neotype strain of *S. pastorianus*, Dunn and Sherlock [45] found three *S. cerevisiae* chromosomes to be lost instead of the eight found by Kodama *et al*., [44] and Rainieri *et al.*, [20]. Proteome profiles obtained by Joubert *et al*. [50], in addition to karyotypes and melibiose degradation in the present study, demonstrate that NRRLY-1551 has been misidentified and should be reclassified as *S. bayanus.* In the ARS collection the *S. pastorianus* neotype is currently strain Y-27171 ( = CBS1538).

### Conclusion

Beer was made by a mixture of yeasts in the past and the development of this technology has led to the formation of several lines of hybrids. Lager beer hybrids have been formerly characterized, and the present study showed that many more beer strains including NBRC1948 or CBS380^T^ are actually mixed lines bearing mosaic genomes related to *S. cerevisiae, S. uvarum* and a pure line closely related to *S. uvarum* which was previously unidentified. We have termed this pure line, *Saccharomyces lagerae* to avoid any confusion with formerly named species. Hybridizations between probable homoploid strains and horizontal transfer(s) might have generated a mosaic genome bearing by *S. bayanus* and the hybrid species *S. pastorianus*. The origins of *S. bayanus*, *S. pastorianus* and their relatedness were summarized as follows. A hybrid *lagerare/uvarum* received genetic material from *S. cerevisiae* probably by horizontal transfer(s). This strain, similar to NBRC1948 (NBRC1948-like), in a backcross with *S. uvarum*, has generated two segregants in each tetrad which were more or less similar to CBS380^T^ and NRRLY-1551. Fertile segregants of NBRC1948-like/*S. uvarum* might have crossed with *S. cerevisiae* wine strains to produce different hybrids constituting the *S. pastorianus* lager strain group. These events have permitted the transfer of *S. cerevisiae* loci -*e.g.* genes of the *MAL* and *MAL*-like containing *MTT1*- into strains of the *S. uvarum* group allowing a better ability to metabolize maltose and maltotriose. In contrast, the cryophilic ability has been very likely gained from the *S. uvarum/lagerae* background.

The most striking fact is how the genomes of these strains have gradually gained their respective contents from the genetic materials of the different species, *Saccharomyces cerevisiae, Saccharomyces uvarum*, and *Saccharomyces lagerae*. The genome sizes measured for *S. bayanus* and *S. pastorianus* were 1.15 and 1.46, respectively [4].

Many cases of interspecific hybrids involving sibling or no sibling species of *S. cerevisiae* were recently described and appear to be rather common [12]. In addition to *S. bayanus* and *S. pastorianus*, hybrids of *S. cerevisiae/S. kudriavzevii* [26] and *S. cerevisiae/S. paradoxus* [51] have been reported.

Finally, beer strains appear as a population of triple or double hybrids descended from three pure lines: *S. uvarum, S. lagerae*, and *S. cerevisiae*. Each strain harbours a set of genes tracing a particular evolutionary route. Hence, studies carried out with different strains using few markers have led to contradictory conclusions. The type strain of the species *S. bayanus* CBS380^T^ is actually a segregant of the back-cross from a first mosaic strain, most likely strain NBRC1948, with *S. uvarum*. This finding leads to a new classification: *S. uvarum* and *S. bayanus* strain CBS380^T^ which are actually a parent/offspring pair. Our proposal to reinstate *S. uvarum* (Beijerinck) as a real species [5] and abolish its synonym or varietal status with *S. bayanus* is fully supported by the data obtained in this study.

The genome of strain CBS7001 and its spore clone 623-6c labelled as *S. bayanus* in data bases should be relabelled as the *S. uvarum* genome [11].

### Additional note

During the revision of our manuscript, the article cited below appeared in PNAS Early Edition in which the authors described a new species named *Saccharomyces eubayanus* whose genome corresponds to the one we named *Saccharomyces lagerae*. Strains *S. bayanus* CBS380^T^ and NBRC1948 are triple hybrids containing sequences from *S. uvarum*, *S. eubayanus* and *S. cerevisiae*.

Microbe domestication and the identification of the wild genetic stock of lager-brewing yeast.

Libkind D., Hittinger C.T., Valério E., Gonçalves C., Diver J., Johnston M., Gonçalves P. and Sampaio J.P. PNAS Early Edition (august 22th).

## Materials and Methods

### Yeast strains

Strains listed in Table 1 are from public collections indicated. Strain *S. uvarum* CBS7001 published by many authors as *S. bayanus* MCYC623, was originally described by Santa Maria as *S. abuliensis* and deposited first in the MCYC (Microbiology Collection of Yeasts Cultures, Madrid, Spain). The MCYC has since ceased to exist, however strains of that collection were integrated into the current CECT (Spanish Type Culture Collection, http://www.springerlink.com/content/h001360t42735684/), but without strain MCYC623 and so can no longer be ordered under the MCYC accession number. Thus, we only retained the CBS number throughout this study. Strains CLIB and NBRC were obtained respectively, from the Collection de Levures d'Intérêt Biotechnologique (INRA, Thiverval-Grignon, France) and NITE Biological Resource Center (NBRC), Japan.

### Media and fermentation tests

YPD medium, growth at 37°C, Mc Clary sporulation medium and fermentation tests were carried out as in [16]. Maltose and maltotriose at final concentration of 1% were used in fermentation tests.

### DNA extraction and molecular techniques

All techniques of DNA extraction, PCR amplification, karyotyping and Southern hybridisation, were performed as previously [5].

### Tetrad analysis and hybrids construction

Tetrads dissections were made under microscope using the micromanipulator de Fonbrune. For each dissection, asci formed on Mc Clary medium were suspended in 25 µl of 120 µg/ml Zymolyase 20T (ICN Biochemicals, Aurora Ohio USA). After 10 min of incubation at 37°C, 50 µl of sterilized water were added to stop the enzyme action. Tetrads were dissected on YPD thin layer which was afterward placed on YPD plate and incubated at 28°C; germinating spores formed visible colonies after three days.

Crosses between NBRC1948 and *S. cerevisiae* tester strain CLIB219 (ade-, red phenotype) or *S. uvarum* CBS7001 were carried out as follows: each strain was allowed to sporulate on Mc Clary at 28°C for 3–4 days, asci were digested with zymolyase as above. One ascus of each parent strain was dissected and two spores, one from each parent were put together by micromanipulation and incubated. After growing at 28°C for 3 days, putative hybrids formed large colonies compared to sporal clones and were purified on YPD. Hybrids between NBRC1948 and *S. cerevisiae* CLIB219 formed white colony, the *ade*- mutation being recessive. Hybrids were confirmed by electrokaryotyping and subsequently allowed to sporulate. Dissection of about 20 tetrads of each cross was carried out to assess fertility. Autodiploidized segregants from F1 tetrads were sporulated to determine the viability of the F2 generation; similar tests were done for the F3 generation.

### Primer selection, PCR amplification and Sequencing of protein-coding genes

For *S. cerevisiae* genes, primers were selected using the GCG Wisconsin package (Genetics Computer Group, Madison, USA) based on the *S. cerevisiae* sequences available for strains S288c, YJM789 and RM11-1a on SGD (http://db.yeastgenome.org) except for the *MAL33* gene: the sequence was from the wine yeast genome EC1118 (Acc N° FN393070). For *S. uvarum* genes the sequences used are from strain CBS7001 ( = MCYC 623), labelled *S. bayanus in* SGD.

For *POL3* of *S. uvarum* strain CBS7001, the gene was fully sequenced (Acc Number FR822194) starting from two non-overlapping contigs AACA01000496 and AACA01000623 in SGD.

PCR amplifications on *S. cerevisiae* and *S. uvarum* genes were carried out as in [16] with a temperature of 50°C for primer hybridization (Tm) except for the *PMA1*, *POL1*, and *POL3* genes of *S. uvarum* for which the Tm was 54°C. Primers used are listed in Table S2. Sequences obtained are deposited in EMBL Nucleotide Sequence Data base, Accession numbers are in Supplementary Table S3.

### Sequence analyses

Sequences were analysed with the Staden package [52] and the GCG Wisconsin package (Genetics Computer Group, Madison, USA). Nucleotide sequence alignments were performed with Clustal [53] included in MEGA5.0 [54]. The evolutionary distances were computed by the neighbor-joining algorithm [55] using the Kimura 2-parameter method also included in MEGA5.0. Phylogenetic trees were visualized with NJ-Plot [56].

### Identification of Saccharomyces lineages by PCR/RFLP of the NTS2 region

The NTS2 of the IGS (rDNA) was amplified and sequenced from *Saccharomyces* strains using primer pair NTSU/ETSL (Table S2); RFLP profiles were determined after digestion with *Alu*I enzyme as previously described [6].

### CGH analyses

Total genomic DNA of CBS7001, CBS380^T^ and NBRC1948 was prepared from cultures grown on YPD. The genomic DNA was labeled and hybridized against GeneChip® yeast genome 2.0 array from Affymetrix (Santa Clara, CA) which covers all *S. cerevisiae* S288C genes, according to Winzeler *et al*. [57]. Labeled fragments were prepared from 200 to 500 ng of genomic DNA using the BioPrime labeling kit (Invitrogen). Hybridization and detection steps were performed at the 'IGBMC Microarray and Sequencing Platform' (Illkirch, France). Two arrays were used for strains CBS380^T^ and CBS7001 and one for NBRC1948. Data were analyzed using the apt 1.12 software: first background was subtracted according to MAS5.0 with apt-cel-Transformer and then intensities for *S. cerevisiae* pm probes extracted with apt-cel-extract. For each pair of arrays, the slope between the two hybridization signals was calculated in order to normalize the signals of the two chips before averaging the hybridization signals of two probes. For each perfect match probe the hybridization ratio between CBS380^T^ (mean of the two arrays) or NBRC1948 and CBS7001 (mean of the two arrays) were calculated with Microsoft Excel which enabled us to score the hybridization intensity all along the genome map. The variations of the hybridization signal were analyzed with CGHScan [58] in order to detect regions with hybridization signals stronger than the background. In some cases CGHScan detected amplified regions separated by less than 1-kb gaps. In such cases, these gaps were interpreted as false negative hybridization, and thus, these regions were considered as contiguous. Telomeric genes were only counted once in the analysis. In the graphs, the log ratios were averaged in an 11 probes sliding window corresponding approximately to one gene.

The *S. cerevisiae* genes encountered in these regions were analyzed with Funspec (http://funspec.med.utoronto.ca/) [59], and categories were filtered with a P value cut-off of 0.01 after Bonferroni correction. All data is MIAME compliant and the raw and transformed data are available at GEO, a MIAME compliant database, under Accession number GSE28225 (http://www.ncbi.nlm.nih.gov/geo/query/acc.cgi?token=tvondwequyqwols&acc=GSE28225).

### S. cerevisiae microsatellite analysis

Amplifications and PCR products were analyzed as described before for *S. cerevisiae* strains [47]. The data obtained for the beer strains were compared to a subset of our former data [47], however, in order to cope with missing data, genetic distances between each strains were computed with MicrosatAnalyzer [60] and in case of aneuploidy one of the supplementary allele was discarded randomly in order to restore diploidy. This did not change the overall topology of the tree in comparison to our former data.

## Supporting Information

Figure S1 **CGH scan of CBS380^T^ and NBRC1948 genomes detecting** ***S. cerevisiae*** **YA,YB and YH fragment.** Graphical representation of the log ratio of the hybridization intensity values for *Saccharomyces bayanus* CBS380^ T^ and NBRC1948 in comparison to *Saccharomyces uvarum* CBS7001 of which the DNA was hybridized against GeneChip® yeast genome 2.0 (Affymetrix). The graphs cover different regions of chromosome where *S. cerevisiae* introgressions A, B and H are revealed.
(TIF)

Figure S2 **Localisation of** ***BDH2*** **and** ***FLO5*** **on chromosomes of** ***S. uvarum*** **and** ***S. bayanus***. A and C. Electrophoretic karyotypes of yeast strains stained with ethidium bromide. B. Probing with *S. cerevisiae BDH2* showing its localisation on the chromosome 1 and its segregation in the tetrad NBCB-2. D. Probing with *S. cerevisiae FLO5* showing its localization on one chromosome of strain NBRC 1948, and on two chromosomes in *S. bayanus, S. uvarum*, *S. carlsbergensis* and *S. cerevisiae*. Arrow heads indicate chromosome 1 of *S. uvarum, S. bayanus* and *S. cerevisiae* hybridized with *S. cerevisiae BDH2* and *FLO5* gene probes. *S. uvarum* chromosomes are numbered according to [6].
(TIF)

Figure S3 **Comparative karyrotypes of strains** ***S. bayanus*** **,** ***S. uvarum*** **,** ***S. pastorianus*** **and** ***S. cerevisiae*** **S288c**. Note 1: Similarity between NBRC539 and NBRC1948 and their difference with NBRC2031. Note 2: Heterogeneity of *S. pastorianus* group: *S. monacensis* CBS1503, *S. carlsbergensis* CBS1513, *S. pastorianus* CBS1538^NT^, NBRC2003. NRRLY-1551 and CBS1538^NT^ exhibit two clearly different chromosomal patterns.
(TIF)

Figure S4 **Evolutionary relationships between NBRC1948 and other** ***S. cerevisiae*** **strains depicted by** ***BIO2*** **and** ***BDH2*** **genes**. The *BIO2* and *BDH2* genes of *S. cerevisiae* strains from various origins were compared. The sequences used are originating from the data published in [16], [24], [25], [34]. The evolutionary history was inferred as for figure 5.
(TIF)

Figure S5 **Chromosomal localisations of** ***MEL1*** **genes in** ***S. uvarum*** **,** ***S. bayanus*** **,** ***S. pastorianus*** **and** ***S. cerevisiae*** **.** A, C. CHEF gels stained with Ethidium bromide. B. Probing with Su*MEL1* gene amplified from *S. uvarum* CBS 395^T^. D. Probing with Sc*MEL1* gene amplified from *S. cerevisiae* ATCC 42637. Crossed hybridization of Sc*MEL1* with CBS395^T^ (*S. uvarum*) and CBS380^T^ (*S. bayanus*) was observed. Arrow heads indicate chromosomes hybridized with each probe.
(TIF)

Figure S6 **Identification of contigs cA, cB, cC by PCR and PCR/RFLP differentiating** ***MAL31*** **/** ***MTY1***. A. Fragment size of *IMA1_MAL33* differentiates contig cA (lower band) in NBCB-6c from cB (upper band) in NBCB-2b. Segregant NBCB-6d and *S. bayanus* strains bearing cB, cA ancC exhibited both PCR bands. B. PCR of *MAL31* or *MTY1* with MAL31yF and specific reversed primers MAL31SpR1 or MTYSpR2. C. *Hinf*1 patterns of *S. cerevisiae MAL31* and *S. carlsbergensis MTY1*. Singles and mixed profiles indicate *MAL31* or *MTY1* as well as both *MAL31* and *MTY1* in different strains *S. bayanus* hybrids. Segregants carrying single copy of *MAL31* or *MTY1* are used as standards.
(TIF)

Figure S7 **Clustering of** ***S. pastorianus*** **in** ***S. cerevisiae*** **according to microsatellite markers analysis.** Neighbor-joining tree showing the clustering of beer isolates among a subset of 140 yeast strains isolated from different sources [24], [47] including the set of sequenced strains of Liti *et al*. [16]. The tree was constructed from the chord distance between strains based on the polymorphism at 12 loci and is rooted according to the midpoint method. Branches are coloured according to the substrate from which strains have been isolated. •Color code: Wine – Europe dark green; Bread yellow; Beer orange; Sake - Japan dark blue; sorghum beer or palm wine - Africa brown; Oak tree - America blue-green; distillery from South America and rum from French Indies purple; Laboratory strains red, Bertram palm – Malaysia blue. Clinical isolates black.
(TIF)

Figure S8 **CARB and UVAR profiles of the NTS2 differentiating** ***Saccharomyces*** **yeasts**. A. NTS2 *Alu*I patterns of *S. bayanus*, *S. uvarum*, *S. carlsbergensis* and *S. cerevisiae*. CARB type pattern of *S. carlsbergensis* is common for *S. bayanus* strain group. NBRC2031 exhibits the UVAR type pattern, while the lager strain NBRC2003 exhibits the *S. cerevisiae* SACE pattern. B. NTS2 *Alu*I patterns of *S. uvarum* CBS7001, *S. bayanus* NBRC1948 and of the hybrid NBCB-10D. Segregation 2∶2 of CARB/UVAR patterns in the tetrads NBCB-6 and NBCB-13. M: marker 1kb plus Invitrogen.
(TIF)

Table S1 **aCGH scan of CBS 380T and NBRC 1948 genomes**
(XLS)

Table S2 **List of primers used**
(XLS)

Table S3 **Accession numbers of nucleotide sequences obtained in this study.**
(XLS)

Table S4 **Identification of** ***S. uvarum*** **nucleotide sequences among 86** ***S. bayanus*** **GSSs in** [**46**] **.**
(XLS)

## References

[1] Corran HS (1975). A history of brewing; Abbot N, editor..

[2] Barnett JA (1992). The taxonomy of the genus *Saccharomyces* Meyen *ex* Reess: a short review for non-taxonomists.. Yeast.

[3] van der Walt JP, Lodder J (1970). *Saccharomyces* Meyen emend. Reess.. The Yeasts, a taxonomic study.

[4] Vaughan-Martini A, Kurtzman CP (1985). Deoxyribonucleic Acid Relatedness among Species of the Genus *Saccharomyces Sensu Stricto*.. International Journal of Systematic Bacteriology.

[5] Nguyen HV, Gaillardin C (2005). Evolutionary relationships between the former species *Saccharomyces uvarum* and the hybrids *Saccharomyces bayanus* and *Saccharomyces pastorianus*; reinstatement of *Saccharomyces uvarum* (Beijerinck) as a distinct species.. FEMS Yeast Res.

[6] Nguyen HV, Lepingle A, Gaillardin CA (2000). Molecular typing demonstrates homogeneity of *Saccharomyces uvarum* strains and reveals the existence of hybrids between *S. uvarum* and *S. cerevisiae*, including the *S. bayanus* type strain CBS 380.. Syst Appl Microbiol.

[7] Kellis M, Patterson N, Endrizzi M, Birren B, Lander ES (2003). Sequencing and comparison of yeast species to identify genes and regulatory elements.. Nature.

[8] Bon E, Neuveglise C, Casaregola S, Artiguenave F, Wincker P (2000). Genomic exploration of the hemiascomycetous yeasts: 5. *Saccharomyces bayanus* var. *uvarum*.. FEBS Lett.

[9] Cliften PF, Hillier LW, Fulton L, Graves T, Miner T (2001). Surveying *Saccharomyces* genomes to identify functional elements by comparative DNA sequence analysis.. Genome Res.

[10] Scannell DR, Zill OA, Rokas A, Payen C, Dunham MJ (2011). The Awesome Power of Yeast Evolutionary Genetics: New Genome Sequences and Strain Resources for the *Saccharomyces sensu stricto* Genus.. G3: Genes, Genomes, Genetics.

[11] Rozpedowska E, Piskur J, Wolfe KH, Kurtzman CP, Fell JW, Boekhout T (2011). Genome sequence of Saccharomycotina: Resources and applications in phylogenomics.. The yeasts, a taxonomic study. fifth edition ed.

[12] Sipiczki M (2008). Interspecies hybridization and recombination in *Saccharomyces* wine yeasts.. FEMS Yeast Res.

[13] Sampaio JP, Goncalves P (2008). Natural populations of *Saccharomyces kudriavzevii* in Portugal are associated with oak bark and are sympatric with *S. cerevisiae* and *S. paradoxus*.. Appl Environ Microbiol.

[14] Gonzalez SS, Barrio E, Querol A (2008). Molecular characterization of new natural hybrids of *Saccharomyces cerevisiae* and *S. kudriavzevii* in brewing.. Appl Environ Microbiol.

[15] Naumov GI (2000). *Saccharomyces bayanus* var. *uvarum* comb. nov., a new variety established by genetic analysis.. Mikrobiologiia.

[16] Liti G, Carter DM, Moses AM, Warringer J, Parts L (2009). Population genomics of domestic and wild yeasts.. Nature.

[17] Naumova ES, Naumov GI, Masneuf-Pomarede I, Aigle M, Dubourdieu D (2005). Molecular genetic study of introgression between *Saccharomyces bayanus* and *S. cerevisiae*.. Yeast.

[18] Masneuf I, Hansen J, Groth C, Piskur J, Dubourdieu D (1998). New hybrids between *Saccharomyces sensu stricto* yeast species found among wine and cider production strains.. Appl Environ Microbiol.

[19] Groth C, Hansen J, Piskur J (1999). A natural chimeric yeast containing genetic material from three species.. Int J Syst Bacteriol 49 Pt.

[20] Rainieri S, Kodama Y, Kaneko Y, Mikata K, Nakao Y (2006). Pure and mixed genetic lines of *Saccharomyces bayanus* and *Saccharomyces pastorianus* and their contribution to the lager brewing strain genome.. Appl Environ Microbiol.

[21] Muller-Wille S (2007). Hybrids, pure cultures, and pure lines: from nineteenth-century biology to twentieth-century genetics.. Stud Hist Philos Biol Biomed Sci.

[22] Liti G, Barton DB, Louis EJ (2006). Sequence diversity, reproductive isolation and species concepts in *Saccharomyces*.. Genetics.

[23] Marinoni G, Manuel M, Petersen RF, Hvidtfeldt J, Sulo P (1999). Horizontal transfer of genetic material among *Saccharomyces* yeasts.. J Bacteriol.

[24] Novo M, Bigey F, Beyne E, Galeote V, Gavory F (2009). Eukaryote-to-eukaryote gene transfer events revealed by the genome sequence of the wine yeast *Saccharomyces cerevisiae* EC1118.. Proc Natl Acad Sci U S A.

[25] Nakao Y, Kanamori T, Itoh T, Kodama Y, Rainieri S (2009). Genome sequence of the lager brewing yeast, an interspecies hybrid.. DNA Res.

[26] Lopes CA, Barrio E, Querol A (2010). Natural hybrids of *S. cerevisiae* x *S. kudriavzevii* share alleles with European wild populations of *Saccharomyces kudriavzevii*.. FEMS Yeast Res.

[27] Pedersen MB (1986). DNA sequence polymorphisms in the genus *Saccharomyces* IV. Homeologous chromosomes III of *Saccharomyces bayanus, S. carlsbergensis,* and *S. uvarum*.. Carlsberg Res Commun.

[28] Stewart GG, Russel I, Spencer JFT, Spencer DM, Smith ARW (1983). Aspects of the biochemistry and genetics of sugar and carbohydrate uptake by yeasts.. Yeast genetics *Fundamental and applied aspects*.

[29] Hinchliffe E, Vakeria D, Walton EF, Yarranton GT (1989). Genetic manipulation of brewing yeasts.. Molecular and cellular cell biology of yeasts: New York: van Nostrand Reinhold.

[30] Salema-Oom M, Valadao Pinto V, Goncalves P, Spencer-Martins I (2005). Maltotriose utilization by industrial *Saccharomyces* strains: characterization of a new member of the alpha-glucoside transporter family.. Appl Environ Microbiol.

[31] Ness F, Aigle M (1995). RTM1: a member of a new family of telomeric repeated genes in yeast.. Genetics.

[32] Muller LA, McCusker JH (2009). A multispecies-based taxonomic microarray reveals interspecies hybridization and introgression in *Saccharomyces cerevisiae*.. FEMS Yeast Res.

[33] Teste MA, Francois JM, Parrou JL (2010). Characterization of a new multigene family encoding isomaltases in the yeast *Saccharomyces cerevisiae*, the IMA family.. J Biol Chem.

[34] Borneman AR, Desany BA, Riches D, Affourtit JP, Forgan AH (2011). Whole-genome comparison reveals novel genetic elements that characterize the genome of industrial strains of *Saccharomyces cerevisiae*.. PLoS Genet.

[35] Alves SL, Herberts RA, Hollatz C, Trichez D, Miletti LC (2008). Molecular analysis of maltotriose active transport and fermentation by *Saccharomyces cerevisiae* reveals a determinant role for the AGT1 permease.. Appl Environ Microbiol.

[36] Dietvorst J, Londesborough J, Steensma HY (2005). Maltotriose utilization in lager yeast strains: MTT1 encodes a maltotriose transporter.. Yeast.

[37] Dietvorst J, Walsh MC, van Heusden GP, Steensma HY (2010). Comparison of the MTT1- and MAL31-like maltose transporter genes in lager yeast strains.. FEMS Microbiol Lett.

[38] Turakainen H, Korhola M, Aho S (1991). Cloning, sequence and chromosomal location of a MEL gene from *Saccharomyces carlsbergensis* NCYC396.. Gene.

[39] Ryu SL, Murooka Y, Kaneko Y (1996). Genomic reorganization between two sibling yeast species, *Saccharomyces bayanus* and *Saccharomyces cerevisiae*.. Yeast.

[40] Naumov GI, Naumova ES, Gaillardin C (1993). Genetic and karyotypic identification of wine *Saccharomyces bayanus* yeasts isolated in France and Italy.. Systematic and applied Microbiology.

[41] Banno I, Kaneko Y (1989). A genetic analysis of taxonomic relation between *Saccharomyces cerevisiae* and *Saccharomyces bayanus*..

[42] Lachance MA (1985). Current views on the yeast species.. Microbiol Sci.

[43] Tamai Y, Tanaka K, Umemoto N, Tomizuka K, Kaneko Y (2000). Diversity of the HO gene encoding an endonuclease for mating-type conversion in the bottom fermenting yeast *Saccharomyces pastorianus*.. Yeast.

[44] Kodama Y, Omura F, Ashikari T (2001). Isolation and characterization of a gene specific to lager brewing yeast that encodes a branched-chain amino acid permease.. Appl Environ Microbiol.

[45] Dunn B, Sherlock G (2008). Reconstruction of the genome origins and evolution of the hybrid lager yeast *Saccharomyces pastorianus*.. Genome Res.

[46] Langkjaer RB, Nielsen ML, Daugaard PR, Liu W, Piskur J (2000). Yeast chromosomes have been significantly reshaped during their evolutionary history.. J Mol Biol.

[47] Legras JL, Merdinoglu D, Cornuet JM, Karst F (2007). Bread, beer and wine: *Saccharomyces cerevisiae* diversity reflects human history.. Mol Ecol.

[48] Galeote V, Bigey F, Beyne E, Novo M, Legras JL (2011). Amplification of a *Zygosaccharomyces bailii* DNA Segment in Wine Yeast Genomes by Extrachromosomal Circular DNA Formation.. PLoS One.

[49] Hansen J, Kielland-Brandt MC (1994). *Saccharomyces carlsbergensis* contains two functional MET2 alleles similar to homologues from *S. cerevisiae* and *S. monacensis*.. Gene.

[50] Joubert R, Brignon P, Lehmann C, Monribot C, Gendre F (2000). Two-dimensional gel analysis of the proteome of lager brewing yeasts.. Yeast.

[51] Zhang H, Skelton A, Gardner RC, Goddard MR (2010). *Saccharomyces paradoxus* and *Saccharomyces cerevisiae* reside on oak trees in New Zealand: evidence for migration from Europe and interspecies hybrids.. FEMS Yeast Res.

[52] Dear S, Staden R (1991). A sequence assembly and editing program for efficient management of large projects.. Nucleic Acids Res.

[53] Larkin MA, Blackshields G, Brown NP, Chenna R, McGettigan PA (2007). Clustal W and Clustal X version 2.0.. Bioinformatics.

[54] Tamura K, Dudley J, Nei M, Kumar S (2007). MEGA4: Molecular Evolutionary Genetics Analysis (MEGA) software version 4.0.. Mol Biol Evol.

[55] Saitou N, Nei M (1987). The neighbor-joining method: a new method for reconstructing phylogenetic trees.. Mol Biol Evol.

[56] Perriere G, Gouy M (1996). WWW-query: an on-line retrieval system for biological sequence banks.. Biochimie.

[57] Winzeler EA, Richards DR, Conway AR, Goldstein AL, Kalman S (1998). Direct allelic variation scanning of the yeast genome.. Science.

[58] Anderson BD, Gilson MC, Scott AA, Biehl BS, Glasner JD (2006). CGHScan: finding variable regions using high-density microarray comparative genomic hybridization data.. BMC Genomics.

[59] Robinson MD, Grigull J, Mohammad N, Hughes TR (2002). FunSpec: a web-based cluster interpreter for yeast.. BMC Bioinformatics.

[60] Dieringer D, Schlötterer C (2003). Microsatellite analyser (MSA): a platform independent analysis tool for large microsatellite data sets.. Molecular Ecology Notes.

[61] Kimura M (1980). A simple method for estimating evolutionary rates of base substitutions through comparative studies of nucleotide sequences.. J Mol Evol.

