# Supplementary figures and images for: Deciphering the Hybridisation History Leading to the Lager Lineage Based on the Mosaic Genomes of Saccharomyces bayanus Strains NBRC1948 and CBS380T
Source: PLoS One. 2011 Oct 5;6(10):e25821. doi: 10.1371/journal.pone.0025821 (PMC3187814; doi:10.1371/journal.pone.0025821)

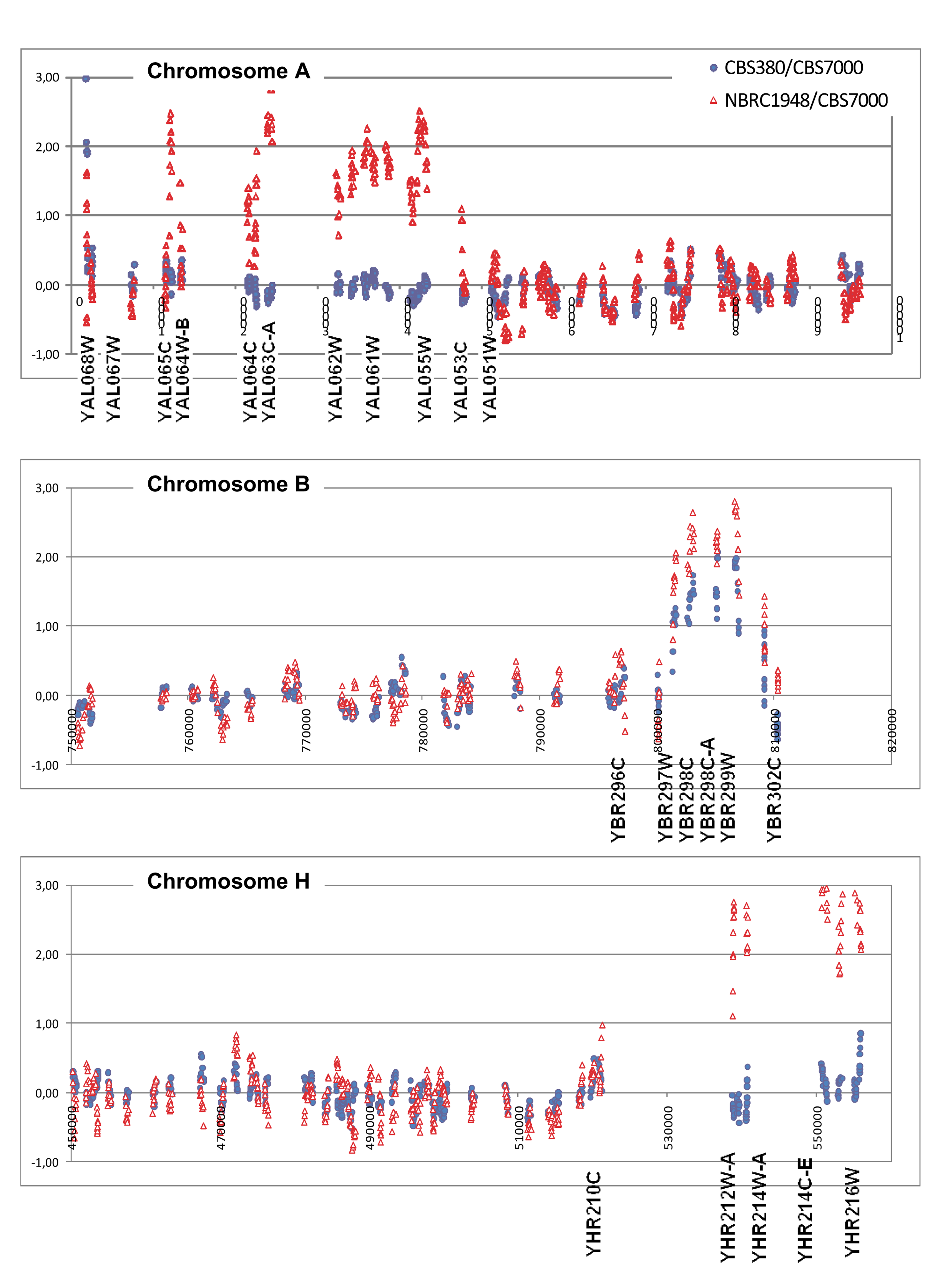

Supplement: Figure S1 — CGH scan of CBS380T and NBRC1948 genomes detecting S. cerevisiae YA,YB and YH fragment. Graphical representation of the log ratio of the hybridization intensity values for Saccharomyces bayanus CBS380 T and NBRC1948 in comparison to Saccharomyces uvarum CBS7001 of which the DNA was hybridized against GeneChip® yeast genome 2.0 (Affymetrix). The graphs cover different regions of chromosome where S. cerevisiae introgressions A, B and H are revealed. (TIF) [file pone.0025821.s001.tif]

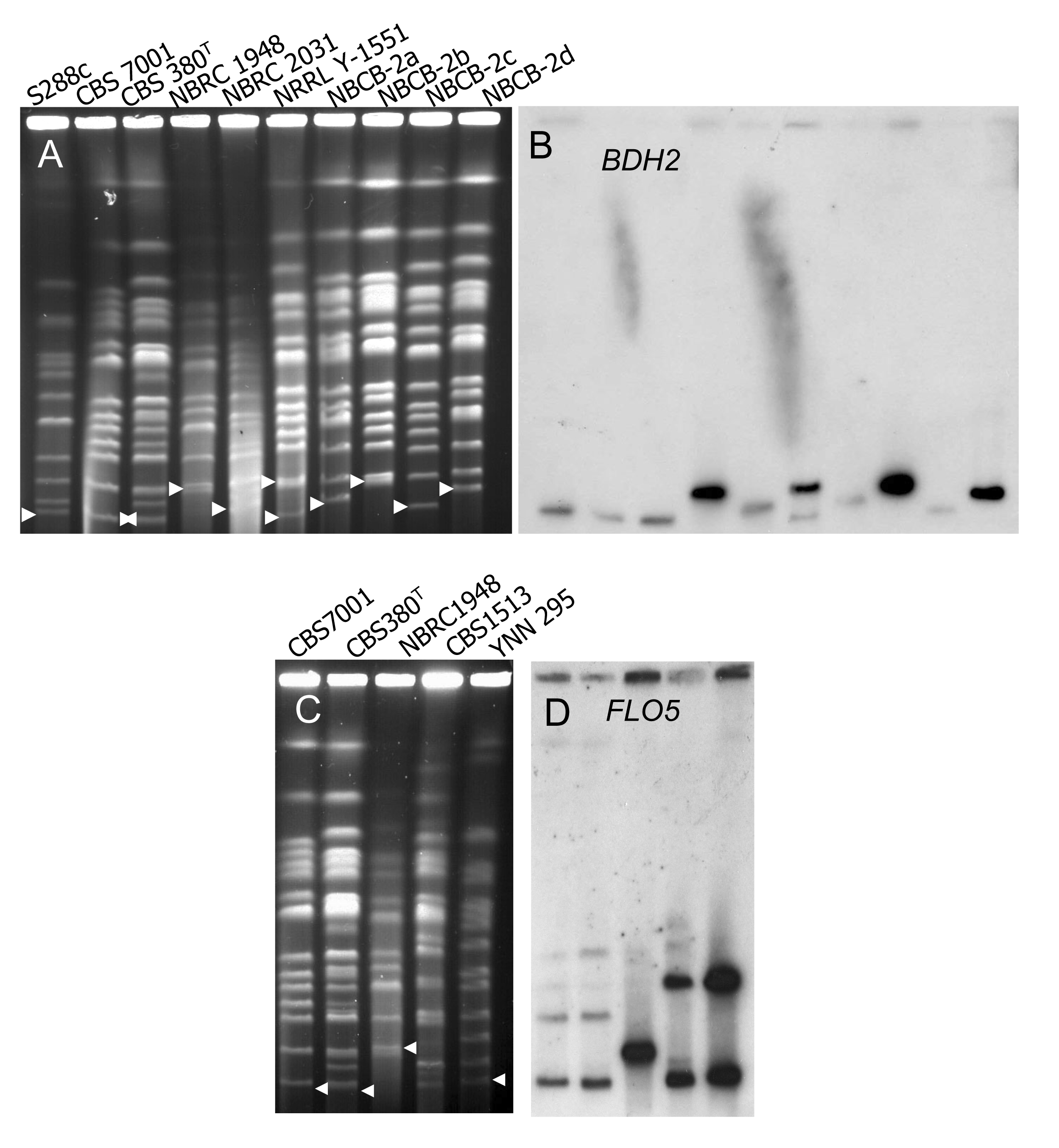

Supplement: Figure S2 — Localisation of BDH2 and FLO5 on chromosomes of S. uvarum and S. bayanus. A and C. Electrophoretic karyotypes of yeast strains stained with ethidium bromide. B. Probing with S. cerevisiae BDH2 showing its localisation on the chromosome 1 and its segregation in the tetrad NBCB-2. D. Probing with S. cerevisiae FLO5 showing its localization on one chromosome of strain NBRC 1948, and on two chromosomes in S. bayanus, S. uvarum, S. carlsbergensis and S. cerevisiae. Arrow heads indicate chromosome 1 of S. uvarum, S. bayanus and S. cerevisiae hybridized with S. cerevisiae BDH2 and FLO5 gene probes. S. uvarum chromosomes are numbered according to [6]. (TIF) [file pone.0025821.s002.tif]

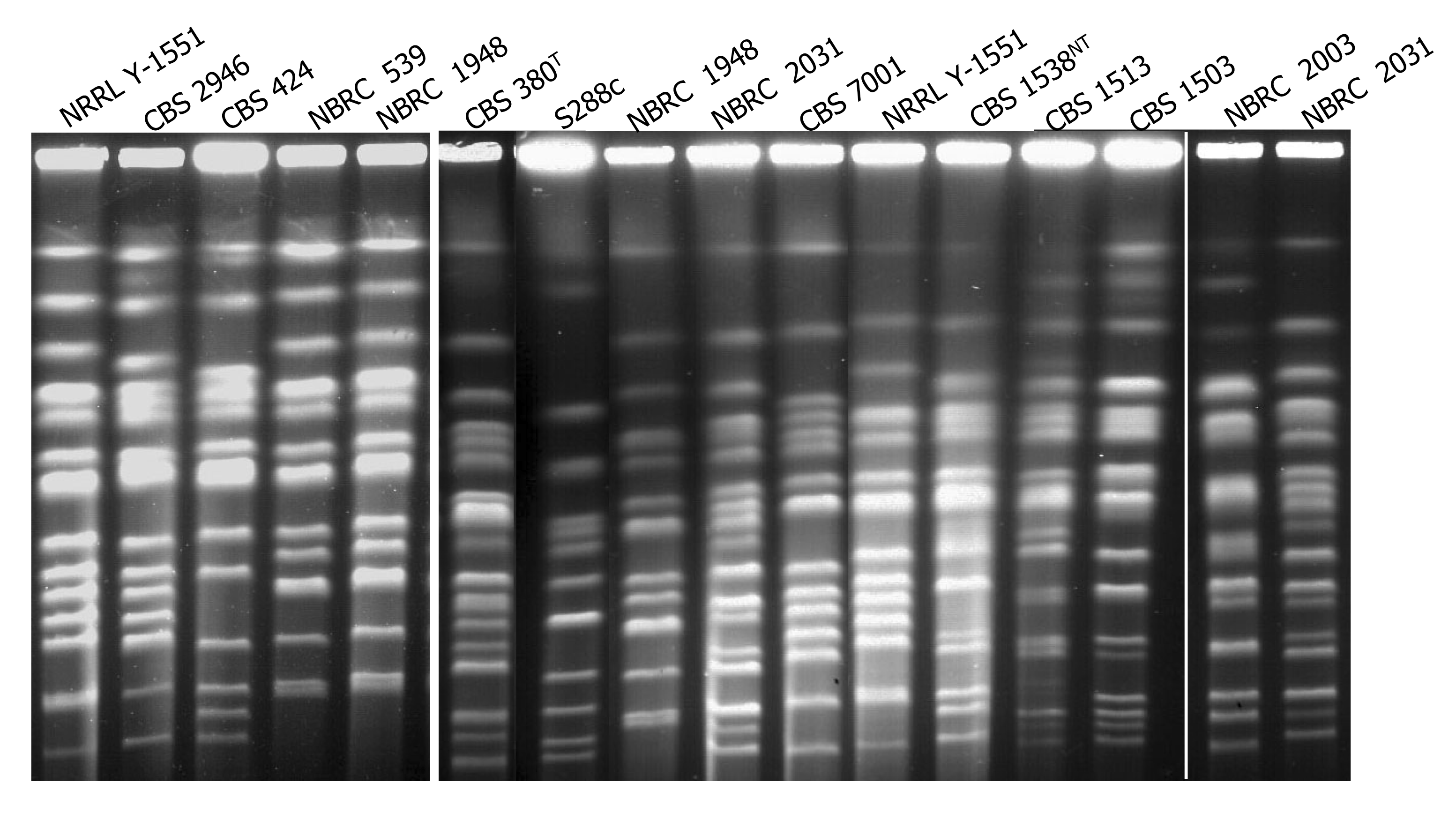

Supplement: Figure S3 — Comparative karyrotypes of strains S. bayanus , S. uvarum , S. pastorianus and S. cerevisiae S288c. Note 1: Similarity between NBRC539 and NBRC1948 and their difference with NBRC2031. Note 2: Heterogeneity of S. pastorianus group: S. monacensis CBS1503, S. carlsbergensis CBS1513, S. pastorianus CBS1538NT, NBRC2003. NRRLY-1551 and CBS1538NT exhibit two clearly different chromosomal patterns. (TIF) [file pone.0025821.s003.tif]

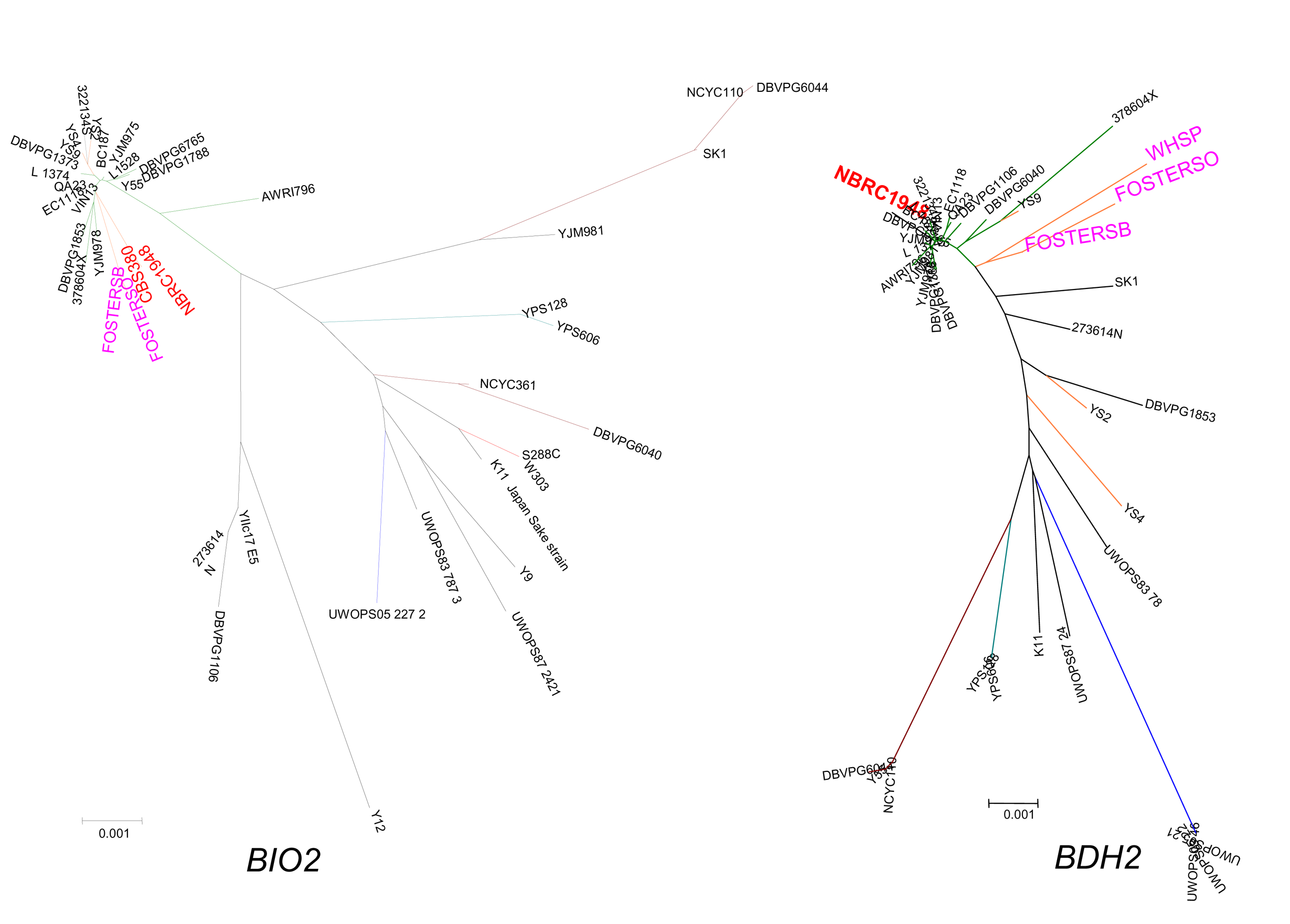

Supplement: Figure S4 — Evolutionary relationships between NBRC1948 and other S. cerevisiae strains depicted by BIO2 and BDH2 genes. The BIO2 and BDH2 genes of S. cerevisiae strains from various origins were compared. The sequences used are originating from the data published in [16], [24], [25], [34]. The evolutionary history was inferred as for figure 5. (TIF) [file pone.0025821.s004.tif]

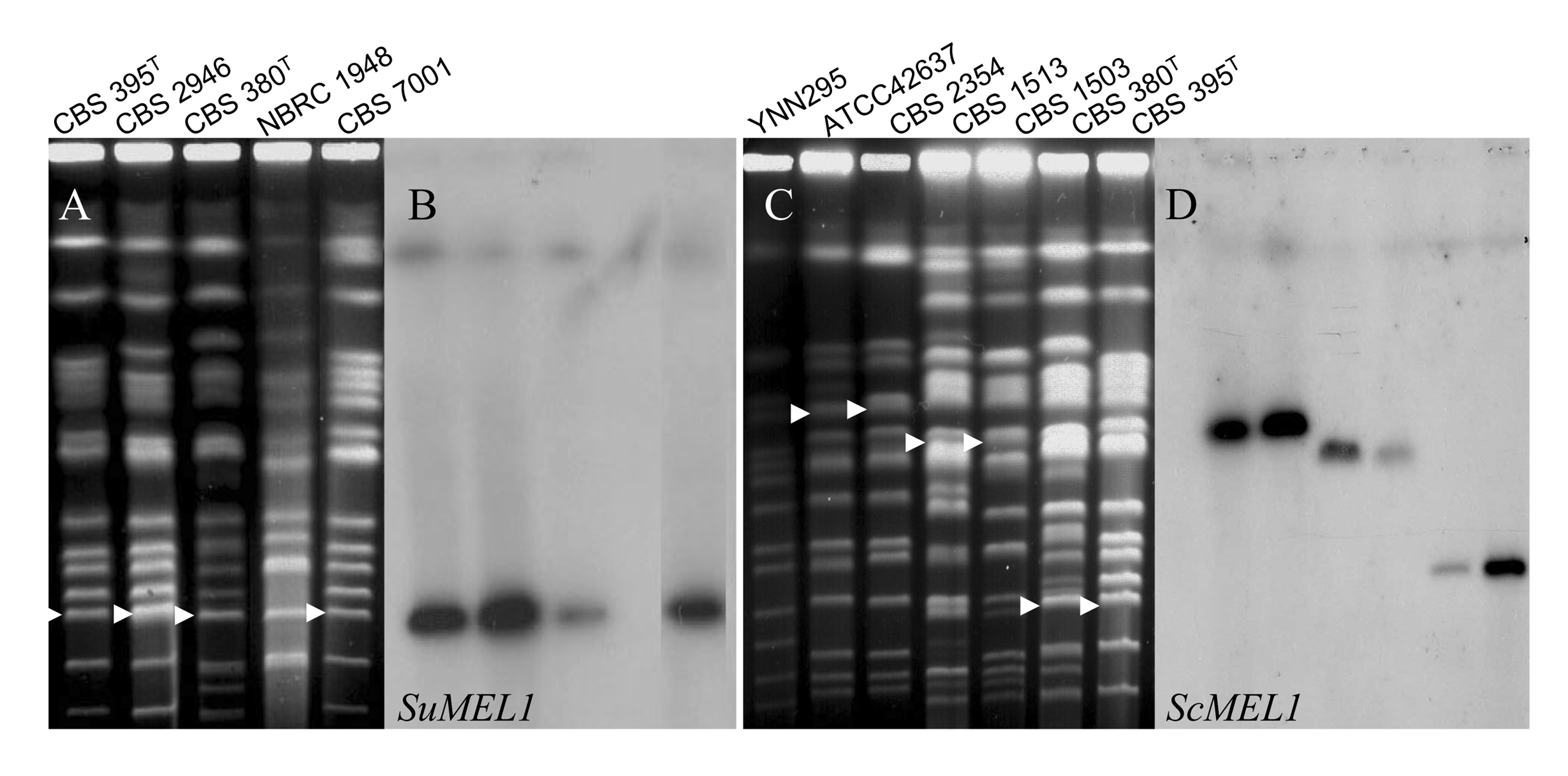

Supplement: Figure S5 — Chromosomal localisations of MEL1 genes in S. uvarum , S. bayanus , S. pastorianus and S. cerevisiae . A, C. CHEF gels stained with Ethidium bromide. B. Probing with SuMEL1 gene amplified from S. uvarum CBS 395T. D. Probing with ScMEL1 gene amplified from S. cerevisiae ATCC 42637. Crossed hybridization of ScMEL1 with CBS395T (S. uvarum) and CBS380T (S. bayanus) was observed. Arrow heads indicate chromosomes hybridized with each probe. (TIF) [file pone.0025821.s005.tif]

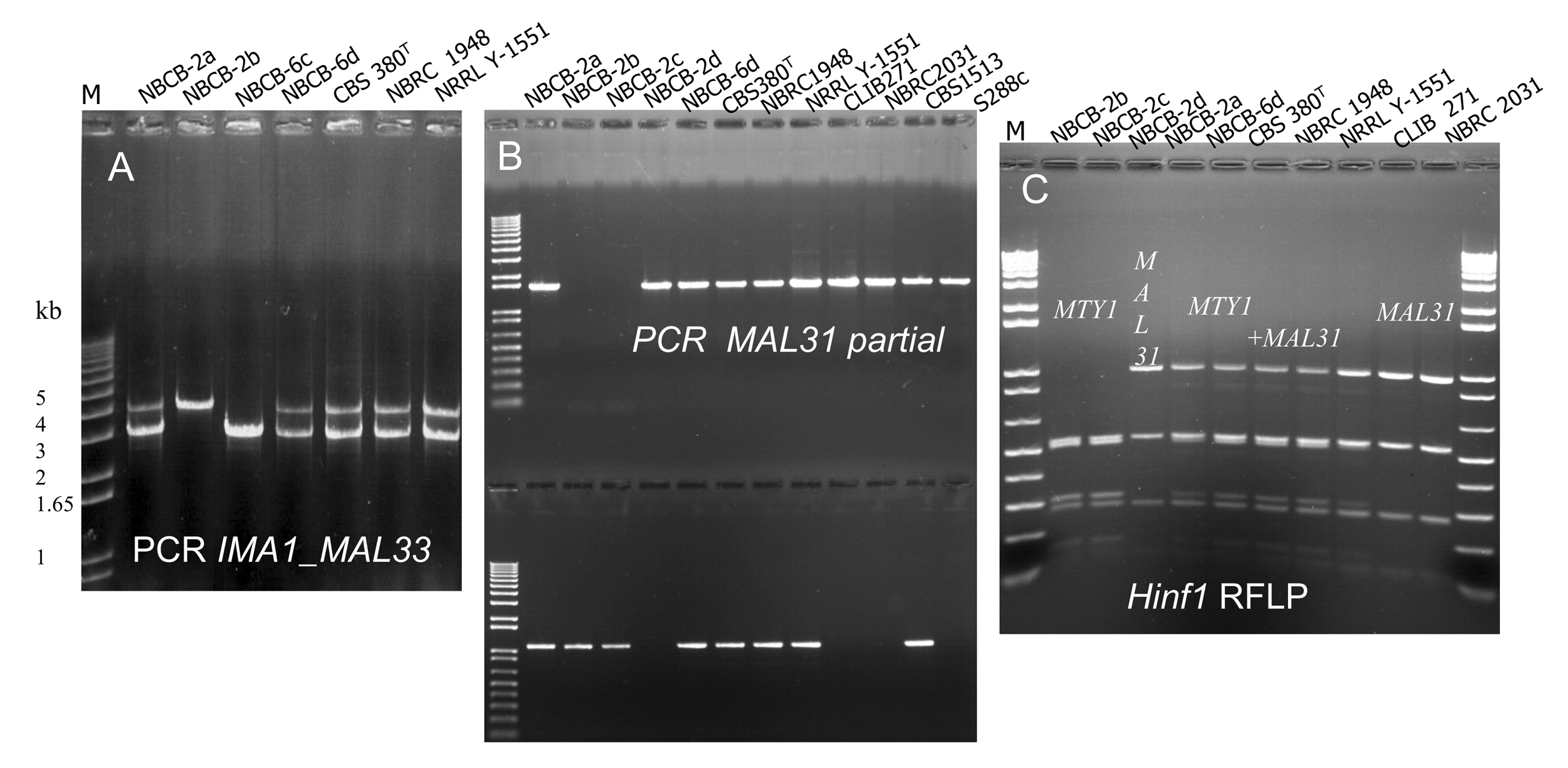

Supplement: Figure S6 — Identification of contigs cA, cB, cC by PCR and PCR/RFLP differentiating MAL31 / MTY1. A. Fragment size of IMA1_MAL33 differentiates contig cA (lower band) in NBCB-6c from cB (upper band) in NBCB-2b. Segregant NBCB-6d and S. bayanus strains bearing cB, cA ancC exhibited both PCR bands. B. PCR of MAL31 or MTY1 with MAL31yF and specific reversed primers MAL31SpR1 or MTYSpR2. C. Hinf1 patterns of S. cerevisiae MAL31 and S. carlsbergensis MTY1. Singles and mixed profiles indicate MAL31 or MTY1 as well as both MAL31 and MTY1 in different strains S. bayanus hybrids. Segregants carrying single copy of MAL31 or MTY1 are used as standards. (TIF) [file pone.0025821.s006.tif]

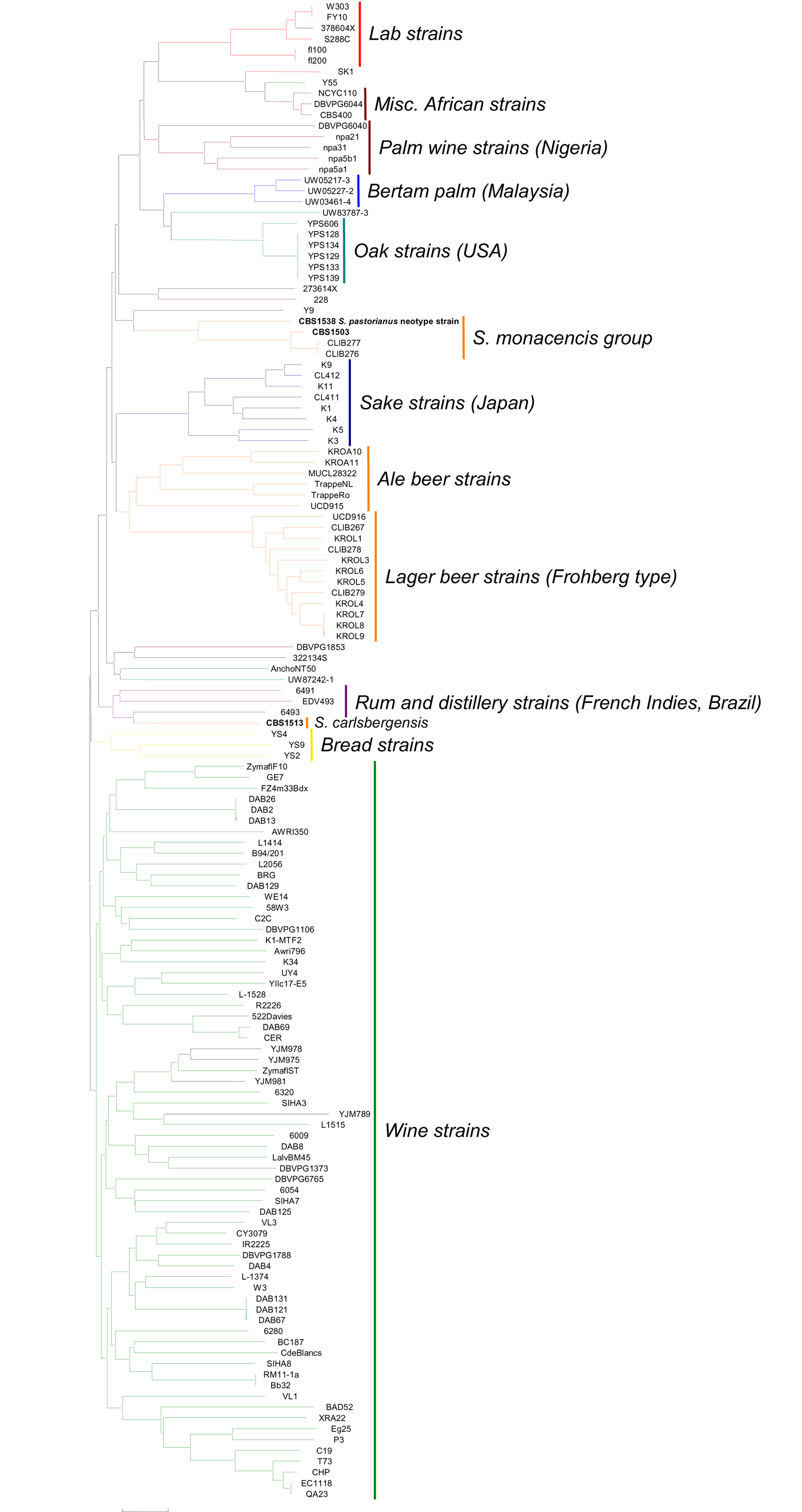

Supplement: Figure S7 — Clustering of S. pastorianus in S. cerevisiae according to microsatellite markers analysis. Neighbor-joining tree showing the clustering of beer isolates among a subset of 140 yeast strains isolated from different sources [24], [47] including the set of sequenced strains of Liti et al. [16]. The tree was constructed from the chord distance between strains based on the polymorphism at 12 loci and is rooted according to the midpoint method. Branches are coloured according to the substrate from which strains have been isolated. •Color code: Wine – Europe dark green; Bread yellow; Beer orange; Sake - Japan dark blue; sorghum beer or palm wine - Africa brown; Oak tree - America blue-green; distillery from South America and rum from French Indies purple; Laboratory strains red, Bertram palm – Malaysia blue. Clinical isolates black. (TIF) [file pone.0025821.s007.tif]

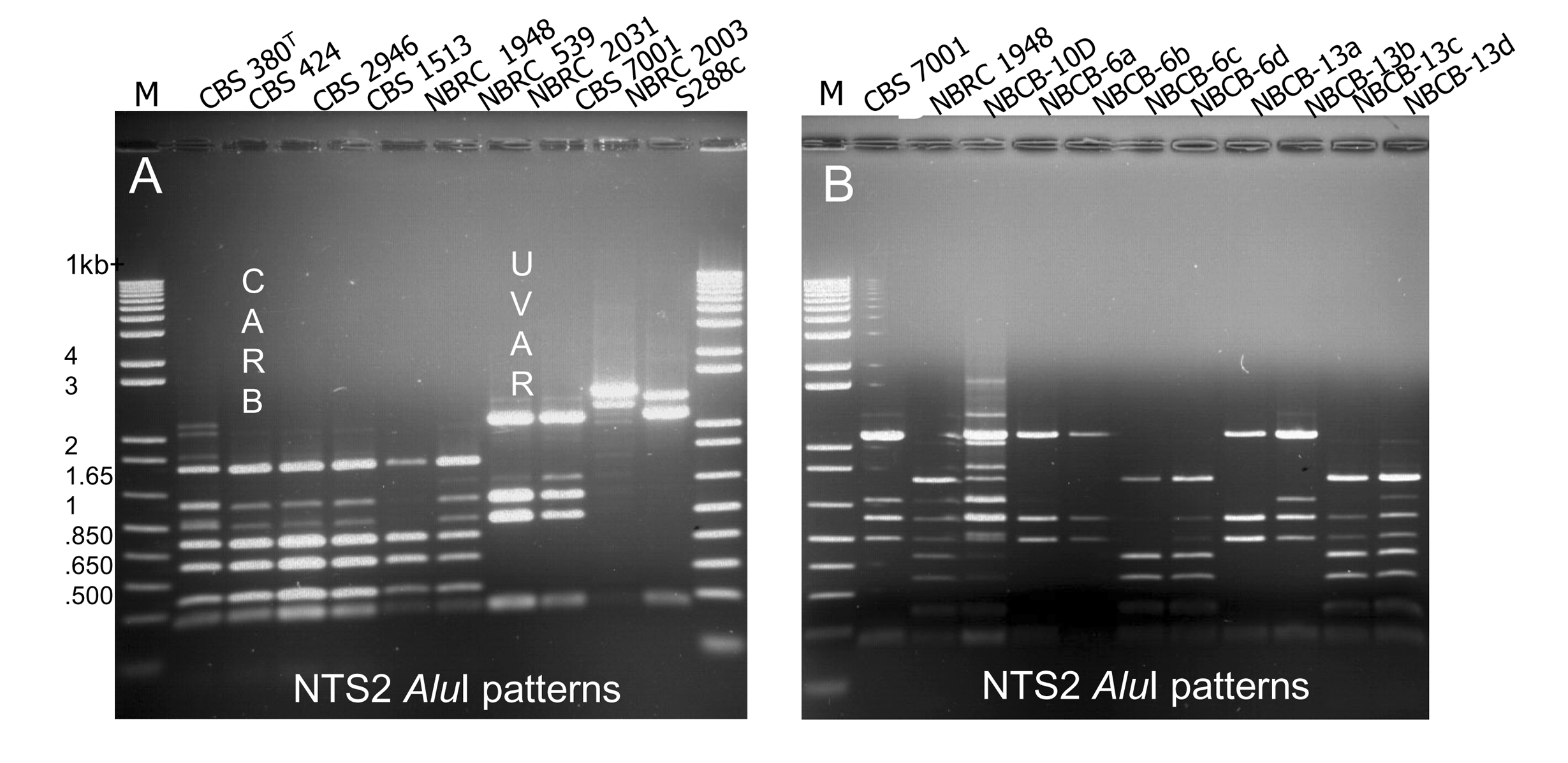

Supplement: Figure S8 — CARB and UVAR profiles of the NTS2 differentiating Saccharomyces yeasts. A. NTS2 AluI patterns of S. bayanus, S. uvarum, S. carlsbergensis and S. cerevisiae. CARB type pattern of S. carlsbergensis is common for S. bayanus strain group. NBRC2031 exhibits the UVAR type pattern, while the lager strain NBRC2003 exhibits the S. cerevisiae SACE pattern. B. NTS2 AluI patterns of S. uvarum CBS7001, S. bayanus NBRC1948 and of the hybrid NBCB-10D. Segregation 2∶2 of CARB/UVAR patterns in the tetrads NBCB-6 and NBCB-13. M: marker 1kb plus Invitrogen. (TIF) [file pone.0025821.s008.tif]
